# Plant-derived cell-penetrating microprotein α-astratide aM1 targets Akt signaling and alleviates insulin resistance

**DOI:** 10.1007/s00018-023-04937-y

**Published:** 2023-09-16

**Authors:** Bamaprasad Dutta, Shining Loo, Antony Kam, James P. Tam

**Affiliations:** 1 School of Biological Sciences, Nanyang Technological University, 60 Nanyang Drive, Singapore, 637551 Singapore; 2 Academy of Pharmacy, Xi’an Jiaotong-Liverpool University, Suzhou, 215123 China; 3 Department of Biological Sciences, Xi’an Jiaotong-Liverpool University, Suzhou, 215123 China

**Keywords:** Leginsulin, Diabetes, α-Astratide, Cell penetration, Microproteins, Glucose uptake, Insulin resistance, Insulin-mimetic, PI3K/Akt signaling, IRS1/2, PKCθ, Cholesterol biosynthesis

## Abstract

Insulin-resistant diabetes is a common metabolic disease with serious complications. Treatments directly addressing the underlying molecular mechanisms involving insulin resistance would be desirable. Our laboratory recently identified a proteolytic-resistant cystine-dense microprotein from huáng qí (*Astragalus membranaceus*) called α-astratide aM1, which shares high sequence homology to leginsulins. Here we show that aM1 is a cell-penetrating insulin mimetic, enters cells by endocytosis, and activates the PI3K/Akt signaling pathway independent of the insulin receptor leading to translocation of glucose transporter GLUT4 to the cell surface to promote glucose uptake. We also showed that aM1 alters gene expression, suppresses lipid synthesis and uptake, and inhibits intracellular lipid accumulation in myotubes and adipocytes. By reducing intracellular lipid accumulation and preventing lipid-induced, PKCθ-mediated degradation of IRS1/2, aM1 restores glucose uptake to overcome insulin resistance. These findings highlight the potential of aM1 as a lead for developing orally bioavailable insulin mimetics to expand options for treating diabetes.

**Supplementary Information:**

The online version contains supplementary material available at 10.1007/s00018-023-04937-y.

## Introduction

Diabetes is a chronic metabolic disease that has become a global epidemic. It affects around 10% of the world's population and consumes up to 20% of global health expenditures [1–3]. Over 90% of diabetes cases are categorized as type 2 diabetes mellitus (T2DM) [3, 4]. T2DM begins with an imbalance between insulin levels and sensitivity that can eventually result in impaired or loss of insulin function. Impaired insulin function in T2DM causes the further manifestation of complications such as glucose intolerance and insulin resistance. Currently, established models for T2DM complications, including insulin resistance, have not adequately explained the mechanisms by which T2DM is initiated and how pathogenesis progresses [5–8]. Although dietary restriction and physical activity can sufficiently manage early-stage T2DM, lifelong treatment with therapeutics that target insulin sensitivity or increase insulin secretion is often needed to prevent complications.

Metformin, a biguanide, is the most widely used first-line treatment for T2DM [9, 10]. Other therapeutics include sulfonylureas, meglitinides, alpha-glucosidase inhibitors, thiazolidinediones, dipeptidyl peptidase IV (DPP-4) inhibitors, and selective amylinomimetics and sodium-glucose transporter-2 (SGLT-2) inhibitors. Often, they are used in combination therapies [10]. These small-molecule T2DM therapeutic agents can have mild-to-serious side effects, including biguanide-induced lactic acidosis, sulfonylurea-mediated cardiovascular complications, DPP-4 inhibitors induced pancreatitis, and SGLT-2 increased risk of urinary and genital tract infections [9–11]. Moreover, antidiabetic drugs such as metformin and thiazolidinediones are contraindicated in patients with cardiovascular and renal complications [9–11]. Thus, therapeutic options with more effective and fewer off-target side effects are needed. Potent and target-selective biologics such as peptide-based antidiabetic therapeutics, including insulin, GLP-1 agonists, gastric inhibitory polypeptide (GIP) agonists, and amylinomimetics have emerged as popular treatment options. However, short biological half-lives and injectable formats pose certain restrictions for their use. Orally bioactive peptides could address some of these limitations. Over the last two decades, our laboratory has focused on identifying novel cysteine-rich peptides (CRPs) from medicinal plants. We focused on a selected group of hyper disulfided CRPs with 6 to 10 cysteine and molecular weights ranging from 2 to 5 kDa [12–20]. These cystine-dense microproteins are structurally compact and highly resistant to chemical and proteolytic degradation, attributes that are required for peptides to be oral bioavailability [12–16]. Importantly, many hyperdisulfided CRP are cell-penetrating and capable of targeting intracellular proteins [12–19].

**Fig. 1:**
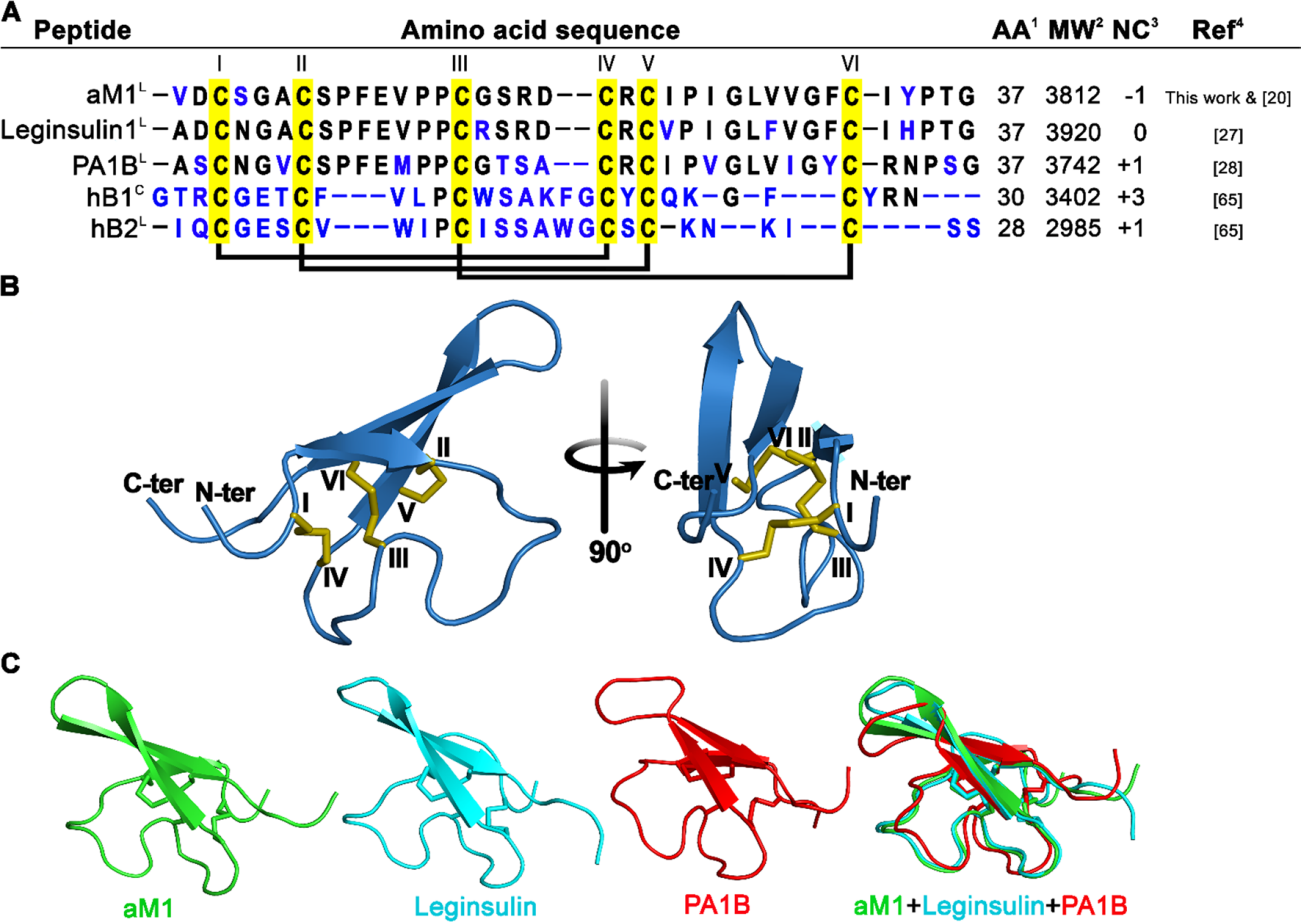
α-astratide aM1 is a cystine-dense microprotein. **A** Comparison of primary sequences and disulfide connectivity of α-astratide aM1 with other reported PA1b-like peptides, including leginsulin1, PA1B, and *Hedyotis biflora* cyclotide hB1 and linear cyclotide hB2. Conserve amino acid residues are represented in black color. ^1^AA: number of amino acids, ^2^ MW: reported mass (Da), ^3^NC: net charge, ^C^Cyclotides and ^L^Linear cyclotides. **B** Model 3D structure for aM1 predicted using the I-TASSER Online Server with the solution structure of leginsulin (PDB: 1JU8) as a template. Disulfide bonds between Cys I-IV, Cys II-V, and Cys III-VI produce a cystine-knot structural fold. **C** Alignment of predicted aM1 structure with leginsulin (PDB: 1JU8) and PA1b (PDB: 1P8B) structures. *T2DM* type 2 diabetes mellitus, *CRP* cysteine-rich peptides, *DMEM* Dulbecco's modified Eagle medium, *FBS* fetal bovine serum, *PBS* phosphate-buffered saline, *LDH* lactate dehydrogenase, *SD* standard deviation, *SEM* standard error of the mean, *INSR* insulin receptor

*Astragalus membranaceus*, commonly known in Chinese as huáng qí (黃芪), is a legume belonging to the Fabaceae family. First documented in Shen Nong's Materia Medica over 2000 years ago, *A. membranaceus* is one of the 50 fundamental herbs used in traditional Chinese medicine (TCM) [21–23]. Huáng qí roots are used as tonifying adaptogenic herbs in TCM and, more recently, have been used to treat diabetes [21–26]. Recently, we reported that two CRP families, α- and β-astratides, exist in huáng qí [20]. β-Astratides have 8 Cys residues and belong to the plant defensins family. In contrast, α-astratide aM1 belongs to the PA1b-like peptide of the leginsulin family [20] and has 37 amino acids with six Cys residues. Similar to other leginsulins, aM1 forms cystine-knot disulfide linkages of Cys I-IV, II-V, and III-VI together with the attendant leginsulin and pea peptide PA1b structural fold (Figs. 1A–C and S3) [20, 27, 28]. However, aM1 shared a low sequence homology with other insulinotropic peptides, including mcIRBP from *Momordica charantia* and conotoxin insulins [29, 30]. Previous studies revealed that leginsulin acts like plant peptide hormones [31, 32]. Recent reports indicated that leginsulin has an insulin-like antidiabetic effect that increases cellular glucose uptake through the insulin signaling pathway [33]. Our previous studies also showed that aM1 modulates β-cell functions and insulin secretion in mouse pancreatic β-cells, suggesting it interferes with glucose homeostasis [20]. However, the mechanism of how aM1 exerts its function on cellular glucose uptake remains elusive.

Here, we report that α-astratide aM1 is a cell-penetrating antidiabetic that enters cells by endocytosis to exert cellular glucose uptake by activating the PI3K/Akt signaling pathway and bypassing insulin-receptor signaling. Combining transcriptomics, antibody arrays, and functional assays, we showed that aM1 represses the expression of genes related to lipid metabolism, reduces intracellular lipid accumulation, and limits lipid-mediated insulin resistance. We also showed that aM1 downregulates PKCθ expression, limits PKCθ-mediated IRS1/2 degradation, and restores IRS1/2 levels in insulin-resistant cells, improving insulin sensitivity in insulin-resistant cells. Our findings are consistent with ethnomedicinal uses for huáng qí and suggest that aM1, which is a plant-derived, stable cystine-dense microprotein of the leginsulin family, represents a promising new class of antidiabetic-insulin substitutes.

## Results

### α-astratide aM1 is a highly stable cystine-dense microprotein

To show that M1 is metabolically stable, we treated aM1 with various conditions simulating oral administration. After a 4 h pepsin digestion at 37 °C in a simulated gastric environment (0.4 mg/ml pepsin in 0.1N HCl buffer, pH 1.2), 98% of aM1 remained intact as determined by reversed-phase HPLC, suggesting that aM1 has good metabolic stability (Fig. S4C). Similar results were obtained with trypsin and chymotrypsin digestion and a 48 h incubation with human serum (Fig. S4A-D). Together, these data indicate that a disulfide scaffold and compact structure can protect aM1 from chemical and enzymatic degradation, which agrees with our previous reports on the high tolerance of cystine-dense peptides to proteolytic degradation [20]. Overall, these data showing that aM1 is likely stable in gastrointestinal and serum environments in vitro may permit its delivery by oral administration.

### α-astratide aM1 is nontoxic

We previously showed that aM1 has insecticidal activity without toxicity up to 200 µM toward mammalian cells [20]. To determine whether aM1 is toxic to cells, we used an MTT-based cell viability assay on multiple cell lines, including C2C12, 3T3-L1, and HEPG2 cells. No substantial changes in cell viability were seen among these three cell lines after being treated with aM1 up to 100 µM dose (Fig. S4E). This result is consistent with our previous studies on CHO-K1 cells that showed no toxicity of aM1 at concentrations up to 200 µM [20]. Additionally, LDH release profiles of aM1-treated cells indicated that aM1 treatment with concentrations up to 100 µM has no significant membranolytic or cytotoxic effects (Fig. S4F). Taken together, aM1 is nontoxic and has no membranolytic, cytotoxic, or mitogenic effects on mammalian cells at concentrations up to 100 µM.

### aM1 is cell penetrating

**Fig. 2:**
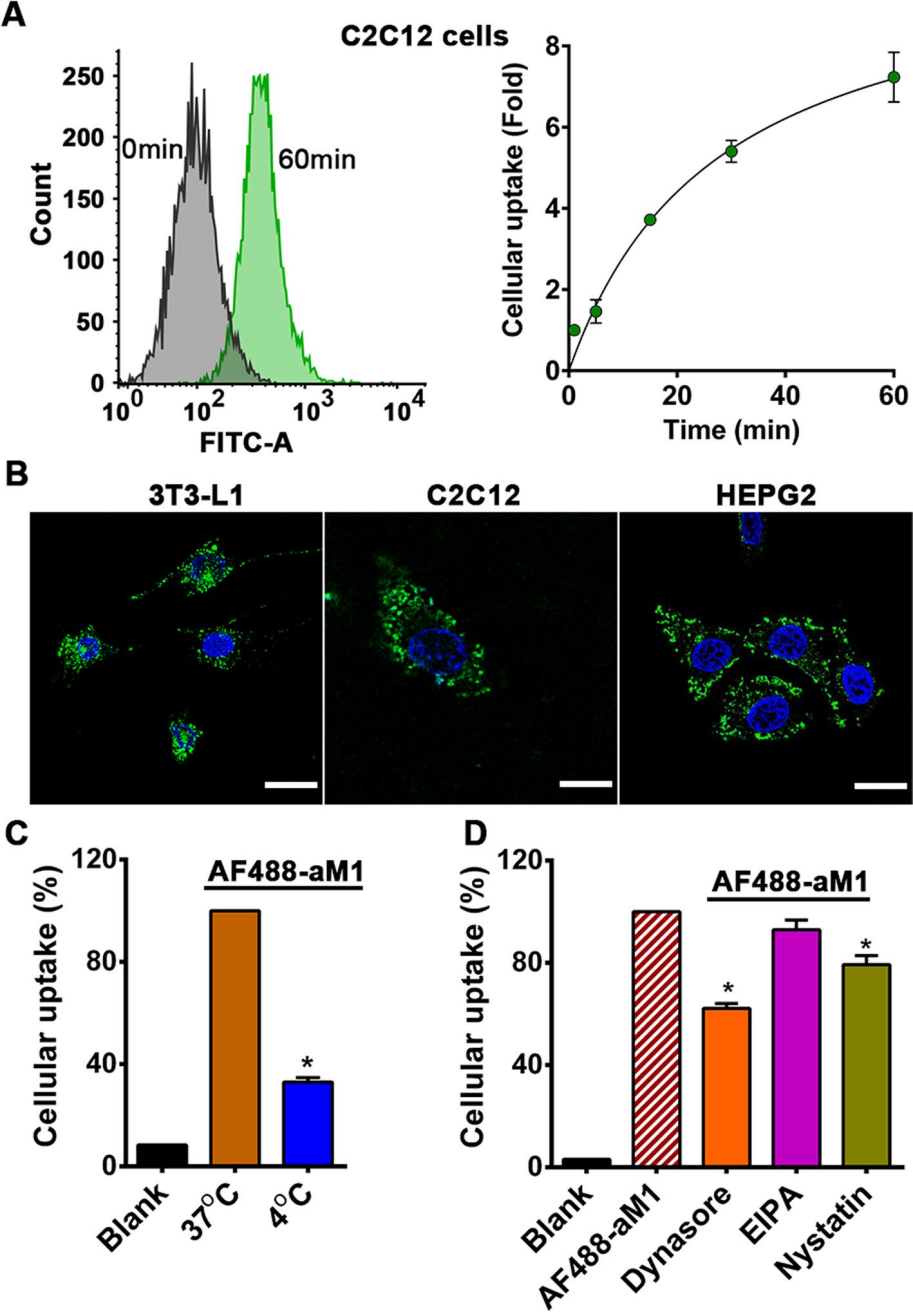
Cellular uptake of AF488-aM1 is endocytosis-dependent. **A** Flow cytometry analysis of C1C12 cells after incubation with 1 μM AF488-aM1 at 37 °C. Data are expressed as fold-change relative to uptake of AF488-aM1 after 1-min incubation. Data are the mean ± SD of three independent experiments. **B** Live-cell confocal microscopy imaging of C2C12, 3T3-L1, and HEPG2 cells after incubation with 1 μM AF488-aM1 at 37 °C. AF488-aM1 is shown in green, and nuclei are counterstained with Hoechst 33342 (blue; scale bar = 20 µm). **C** Flow cytometry analysis of C1C12 cells after pre-incubation at 4 °C for 30 min before incubation with 1 μM AF488-aM1 at 4 °C for 1 h. Control cells were incubated with 1 μM AF488-aM1 at 37 °C for 1 h. **D** Flow cytometry analysis of C2C12 cells pretreated for 30 min with different endocytosis inhibitors, including 50 µM dynasore, 50 μM ethyl isopropyl amiloride (EIPA), and 50 μg/ml nystatin, followed by incubation with 1 μM AF488-aM1 for 1 h at 37 °C. Data are shown as mean values (± SD), and statistical significance was calculated (analysis of variance (ANOVA) with Dunnett’s multiple comparison test) from three independent experimental replicates. *p < 0.05 compared to control

With a net negative charge, aM1 is hydrophobic with a 75% sequence containing hydrophobic amino acid residues, which include six Cys, five Gly, five Pro, and seven Val and Ile. Previously, we showed that cystine-rich microproteins or Pro-rich peptides are cell-penetrating [15, 17, 34]. To show that aM1 is cell-penetrating, we labeled site-specifically its N terminus with Alexa Fluor 488. Dye-labeled aM1 (AF488-aM1) was purified by RP-HPLC, and its identity was confirmed by MALDI-TOF MS (Fig. S5A, B). Cellular uptake of AF488-aM1 by C2C12 mouse myoblasts was quantified by flow cytometry. Incubation with 1 μM AF488-aM1 significantly increased the fluorescence intensity of the treated cells, and the intensity reached a plateau in 1 h (Fig. 2A, B). Confocal microscopy-based live-cell imaging was used to minimize artifacts in the localization of labeled peptides in fluorescence microscopy [12, 15]. Live-cell imaging of C2C12, 3T3-L1, and HEPG2 cells after incubation with 1 μM AF488-aM1 for 1 h showed that AF488-aM1 was internalized and distributed throughout the cytoplasm without nuclear accumulation (Fig. 2B). Similar results were obtained in three different cell types (Fig. 2B), indicating that the cellular uptake of AF488-aM1 is cell-type independent.

To understand the cell-penetrating mechanism of aM1, cells were treated with 1 μM AF488-aM1 for 1 h, each at 4 °C and 37 °C. Analysis by flow cytometry showed a significant reduction in aM1 uptake at 4 °C compared to 37 °C (Fig. 2C), indicating energy-dependent endocytosis-mediated uptake. To further assess endocytosis-mediated aM1 uptake, cells were pre-incubated with different endocytosis inhibitors for 30 min before exposure to aM1 for 1 h. Dynasore-mediated inhibition of dynamin-dependent endocytosis significantly lowered aM1 uptake, confirming the involvement of clathrin-mediated endocytosis (Fig. 2D). In addition, flow cytometric analysis suggests no significant involvement of receptor- and caveolin-mediated endocytosis in aM1 internalization (Fig. 2D). Together, our data showed that aM1 is cell-penetrating and enters various cell types by endocytosis.

### aM1 promotes cellular glucose uptake

**Fig. 3:**
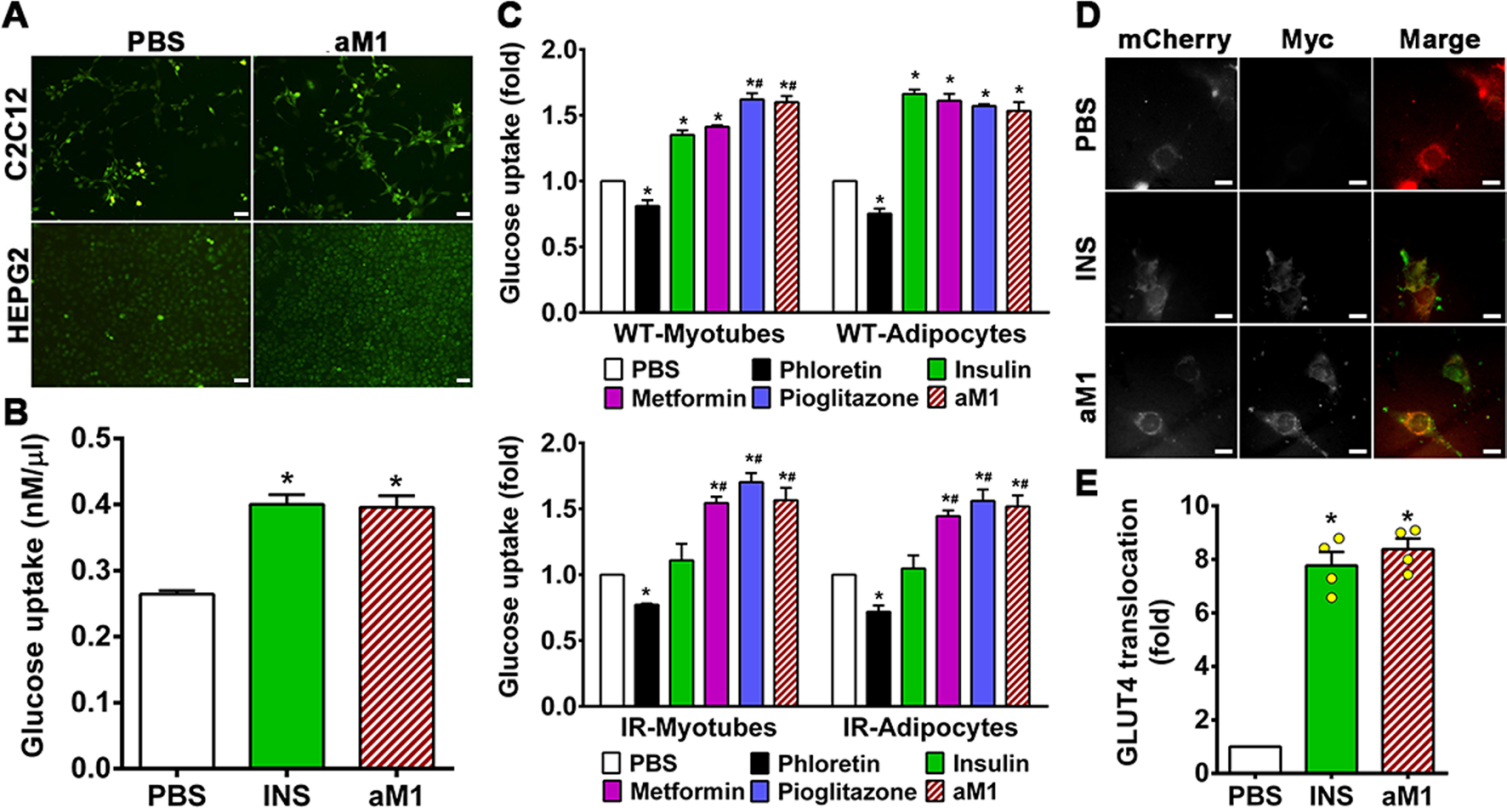
α-Astratide aM1 promotes cellular glucose uptake. **A** C2C12 and HEPG2 cells were cultured with or without aM1 for 24 h, and cellular glucose uptake was observed by fluorescence microscopy following the uptake of 2-NBDG (scale bar = 50 µm). **B** Glucose uptake of PBS or 100 nM insulin (INS) or 20 µM aM1-treated C2C12 cells in 1 h. Glucose consumption was estimated by depleting glucose levels of the growth medium using a glucose assay kit. Data presented the mean ± SEM (*n* = 3, analysis of variance (ANOVA) with Tukey's multiple comparisons test). *p < 0.05 versus PBS control. **C** aM1 increases cellular glucose uptake in wild-type (WT) and insulin-resistant (IR) cells. Relative glucose uptake was measured in wild-type (top) and insulin-resistant (bottom) C2C12-myotubes and 3T3-L1-adipocytes using a 2NBDG uptake assay to estimate the glucose consumption of cells treated with the respective compounds. Three independent experimental replicates represent the mean ± SD of relative glucose uptake (ANOVA with Tukey's multiple comparisons test). *p < 0.05 versus PBS-control groups, and ^#^*p* < 0.05 versus insulin-treated groups. **D** aM1-stimulated GLUT4 surface exposure in C2C12-myotubes. Serum-starved cells were stimulated with 100 nM insulin (INS) for 30 min and 20 μM aM1 for 24 h, with PBS as a control. Surface GLUT4 was detected and quantitated using anti-Myc fluorescence (Alexa Fluor 488) immunolabelling of nonpermeabilized cells. Data are representative microscopic images (scale bar = 15 µm). **E** GLUT4 translocation was assessed using Myc fluorescence intensity. Data for each group are expressed as relative fold-change relative to PBS-treated control cells. Data are d mean values (± SD) of about 50 cells in each experimental condition from three independent experiments (ANOVA with Tukey's multiple comparisons test). *p < 0.05 vs. PBS control

To show that aM1 is antidiabetic, we performed a 2-NBDG-uptake assay to examine how aM1 affects cellular glucose uptake in different cell lines. Mouse myoblast C2C12 cells and human hepatocyte HEPG2 cells treated with 20 µM aM1 exhibited a 50% increase in cellular glucose uptake compared to vehicle control (Fig. 3A and S6A). In addition, we performed a colorimetric measurement of glucose in the culture medium to validate our 2NBDG-based glucose uptake results. Consistent with our findings, the colorimetric results also revealed a 1.5-fold increase in glucose consumption by C2C12 cells in the presence of 20 µM aM1 compared to untreated control cells (Fig. 3B). The effects of aM1 were similar to those seen for 100 nM insulin treatment, which acted as a positive control (Fig. 3B). Overall, these results suggest that aM1 has promising antidiabetic activity but may be less potent than insulin at the tested doses.

### α-Astratide aM1 promotes in vitro peripheral glucose uptake

About 33% of blood glucose is taken up through insulin-sensitive peripheral tissues, including muscles and adipose tissue. This peripheral glucose uptake plays a central role in maintaining glucose homeostasis in the body. The pathophysiological effects of T2DM significantly compromise peripheral glucose uptake. To explore how aM1 affects peripheral glucose uptake, we established C2C12 myotube- and 3T3-L1 adipocyte cell line-based in vitro models [35, 36]. Cellular glucose uptake was measured using the fluorescently labeled glucose derivative, 2-NBDG, with 100 nM insulin, 20 µM metformin, and pioglitazone used as a positive control. Fluorometric measurement showed a 1.4-fold increase in glucose uptake by wild-type C2C12-myotubes treated with 20 µm aM1 (Fig. 3C, top panel). Treatment with 100 nM insulin and 20 µM metformin/pioglitazone had a comparable effect on glucose uptake (Fig. 3C, top panel). Cell-based glucose uptake studies reflected a dose-dependent response of aM1. Treatment of wild-type 3T3-L1 adipocytes with 20 µM aM1 increased glucose uptake by 1.5-fold over vehicle-treated control cells (Fig. 3C, top panel). The 2-NBDG uptake assay confirmed the antidiabetic effects of aM1 and its ability to boost peripheral glucose uptake.

### α-astratide aM1 improves glucose uptake in insulin-resistant cells

Insulin resistance is a common pathological feature of T2DM wherein insulin-sensitive peripheral tissues, including muscles and adipose tissue, do not respond to insulin stimulation. Insulin resistance compromises peripheral tissue functionality by reducing glucose uptake efficiency and increasing blood glucose levels. We assessed the efficacy of aM1 peptides in insulin-resistant cells that we established by exposing the cells to a medium containing high (100 nM) insulin concentration. Indeed, insulin-resistant C2C12-myotubes and 3T3-L1-adipocytes cells resisted insulin stimulation, and glucose uptake remained at basal levels after insulin treatment (Fig. 3C, bottom panel). Treatment of insulin-resistant cells with aM1 significantly increased glucose uptake (1.5-fold) relative to untreated cells, which retained glucose at the basal levels (Fig. 3C, bottom panel). This increase was similar to the 1.5- and 1.6-fold increase in glucose uptake for insulin-resistant myotubes and adipocytes treated with metformin and pioglitazone, respectively (Fig. 3C, bottom panel). Collectively, these results show that aM1 has the potential to mitigate insulin resistance and enhance glucose uptake in peripheral tissues.

### α-astratide aM1 increases GLUT4 translocation

In skeletal muscles and adipocytes, insulin-induced glucose uptake is predominantly mediated by GLUT4 glucose transporters. Thus, we examined how aM1 treatment affects GLUT4 translocation. We determined GLUT4-mediated glucose uptake using a stable C2C12 cell line expressing Myc-GLUT4-mCherry fusion protein that has a Myc epitope attached to the first N-terminal exofacial loop in GLUT4 and a mCherry fusion at the GLUT4 C terminus that allows detection of membrane-embedded GLUT4 under nonpermeabilized conditions (Fig. S6D) [37–39]. Confocal microscopy revealed a significant increase in the intensity of green fluorescence in aM1-treated cells under nonpermeabilized conditions compared to the PBS control group (Fig. 3D, E). This increase in fluorescence indicates a substantial increase in the surface presence of GLUT4 after aM1 treatment. Furthermore, the increased amounts of GLUT4 at the cell surface were observed after insulin stimulation (Fig. 3D, E). These findings suggest that aM1 treatment promotes GLUT4 translocation to the cell surface, resulting in enhanced GLUT4-mediated glucose uptake in C2C12 cells.

### Cell-penetrating aM1 activates the Akt signaling pathway

**Fig. 4:**
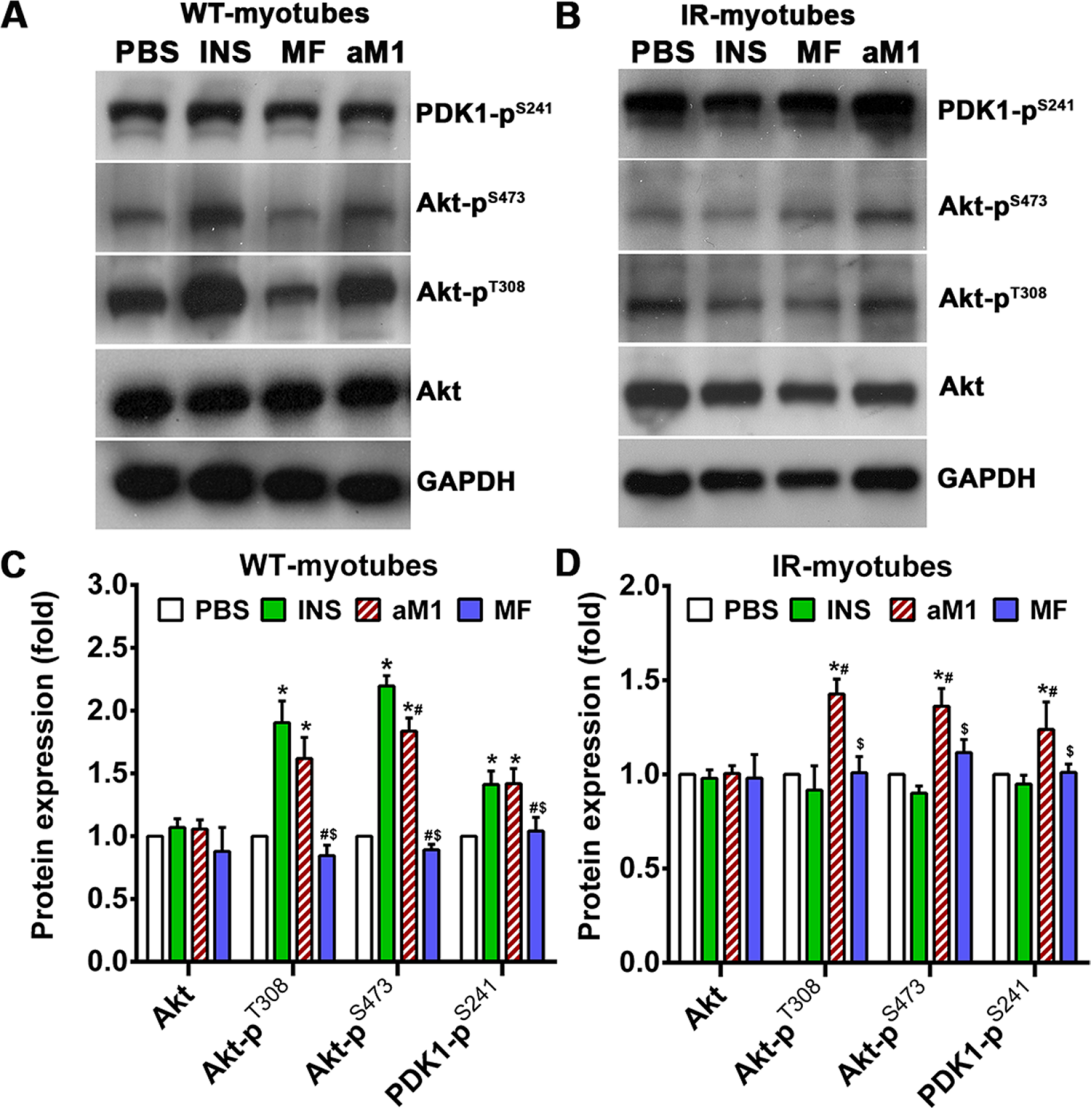
aM1 activates the PI3K/Akt pathway in C2C12-myotubes. Normal proximal PI3K/Akt signaling in C2C12-myotubes. Cells were treated for 24 h with mock (PBS), aM1, or metformin (MF) or with insulin (INS) for 30 min. **A** Activation of PI3K/Akt signaling leads to phosphorylation of PDK1 at S241 and Akt at T308 and S473 in wild-type (WT) C2C12-myotubes. **B** In insulin-resistant (IR) C2C12-myotubes, aM1 activates PI3K/Akt signaling and phosphorylates PDK1 and Akt via an insulin-independent mechanism. **C**–**D** Data in each group were normalized with respect to GAPDH expression and stated as fold-change relative to PBS control cells for WT C2C12-myotubes (panel **C**) and IR C2C12-myotubes (panel **D**). Data show corresponding mean ± SD of three independent experiments (analysis of variance (ANOVA) with Tukey's multiple comparisons test). *p < 0.05 versus PBS-control groups, ^#^*p* < 0.05 versus INS-treated groups and ^$^*p* < 0.05 versus aM1-treated groups

Glucose is transported into cells through glucose transporter (GLUT) proteins, while in peripheral tissue, GLUT4 isoforms are the primary glucose transporter [40]. GLUT4 translocation to the plasma membrane is mediated by protein kinase B/Akt, a crucial member of the Akt signaling pathway that facilitates insulin-dependent glucose transport in peripheral tissue [41–43]. Activating Akt requires phosphorylation of Akt at Thr308 and Ser473 [44]. Western blot analysis was performed to evaluate the impact of aM1 on Akt signaling, and it showed that aM1 treatment did not significantly affect GLUT4 expression in either C2C12-myotubes or 3T3-L1-adipocytes (Fig. S6C). However, Akt (protein kinase B) phosphorylation at Thr 308 and Ser473 was increased by 1.6-fold and 1.8-fold, respectively, in aM1-treated wild-type C2C12 myotubes compared to the control group (Fig. 4A, C). Wild-type 3T3-L1 adipocytes showed a 1.5-fold increase in Akt phosphorylation at Thr308 and Ser473 relative to untreated cells (Fig. S7A, C). After insulin stimulation, the expected Akt phosphorylation was observed in wild-type myotubes (Fig. 4A–C) and adipocytes (Fig. S7A, C). In wild-type myotubes treated with metformin, the amount of phosphorylated Akt remained at basal levels (Fig. 4A, C). Although no significant differences were observed for total Akt expression between untreated cells or those treated with aM1 or insulin (Fig. 4A, C and S7A, C), a significant increase in PDK1 phosphorylation at Ser242 was detected after insulin and aM1 treatment of wild-type cells (Fig. 4A, C and S7A, C). At the same time, no significant effect was found after metformin treatment (Figs. 4A, C, S7A, C). Therefore, the western blot analysis indicates that aM1 activates the Akt signaling cascade in the wild-type cells.

**Fig. 5:**
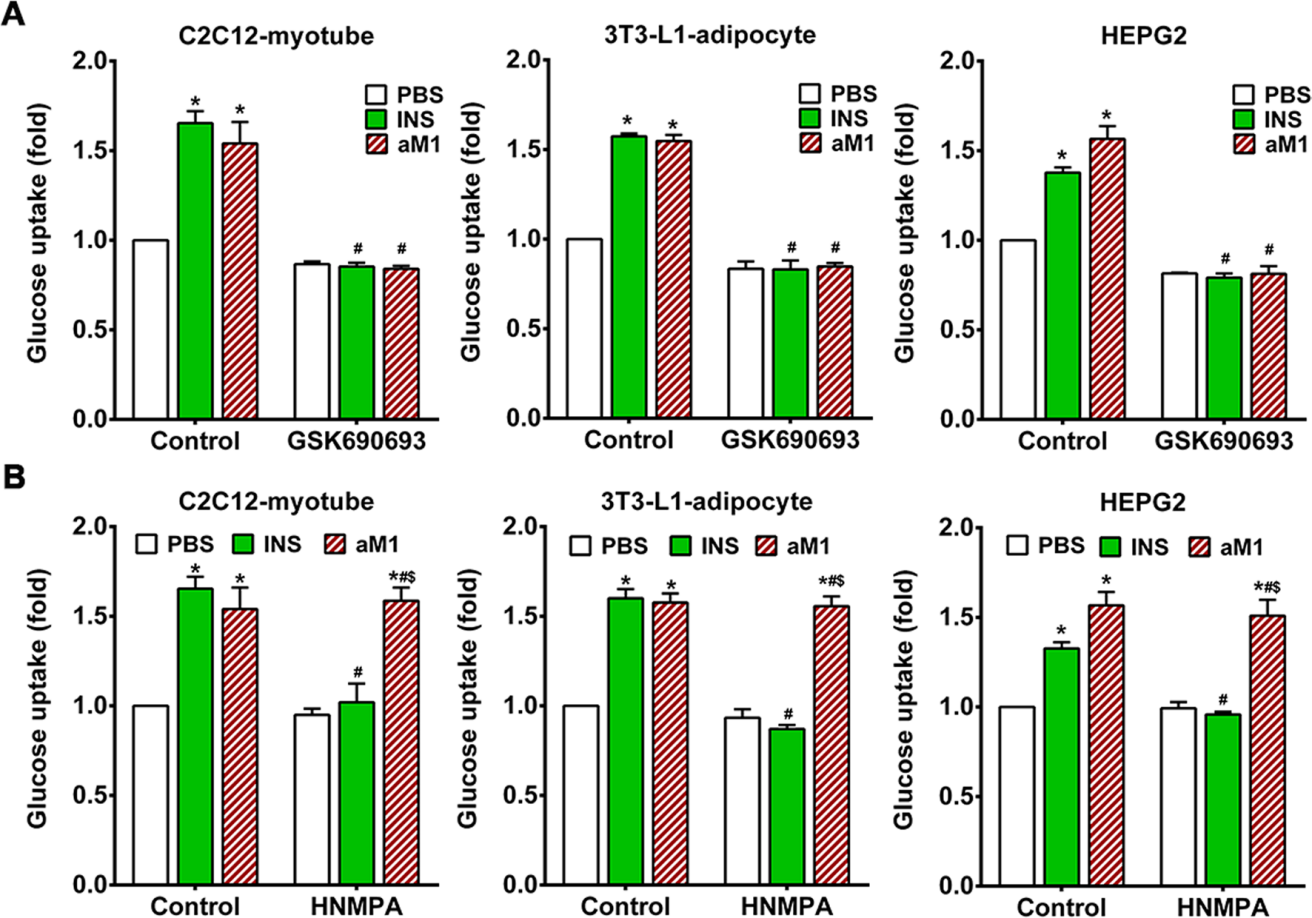
aM1 activates Akt-mediated glucose uptake in an insulin receptor activation signal-independent manner. **A** Relative glucose uptake of C2C12-myotubes, 3T3-L1-adipocytes, and HEPG2 in the presence or absence of Akt-inhibitor GSK690693 and co-treatment with 100 nM insulin (INS) for 30 min or 20 µM aM1 (24 h pretreated). **B** Relative glucose uptake of C2C12-myotubes, 3T3-L1-adipocytes, and HEPG2 cells that were treated or not with the insulin receptor inhibitor HNMPA and were co-treated or not with 100 nM insulin (INS) for 30 min and 20 µM aM1 (24 h pretreated). The relative glucose uptake in each group is expressed as fold-change relative to the respective PBS-treated control groups. Data represent mean values (± SD) from three independent experimental replicates (analysis of variance (ANOVA) with Sidak's multiple comparisons test). *p < 0.05 versus PBS-treated control groups, ^#^*p* < 0.05 versus INS-treated control groups and ^$^*p* < 0.05 versus INS-treated HNMPA groups

To further validate this finding, glucose uptake was assessed in the presence of the Akt inhibitor GSK690693. Co-treatment of C2C12 myotubes, 3T3-L1 adipocytes, and HEPG2 cells with GSK690693 inhibited both insulin- and aM1-mediated glucose uptake (Fig. 5A). Taken together, these results support our findings and strongly suggest that aM1-mediated glucose uptake is dependent on Akt signaling, which is consistent with the reported molecular events involved in insulin-dependent glucose uptake [42–44].

### aM1 activates the Akt signaling pathway in an insulin receptor-independent manner

The PI3K/Akt signaling pathway is an integral part of the insulin-dependent glucose uptake pathway, and phosphorylation of insulin receptor (INSR) upon insulin binding initiates a phosphorylation cascade that activates the Akt signaling cascade, ultimately leading to GLUT4 translocation to the plasma membrane [45, 46]. However, insulin resistance disrupts INSR-mediated tyrosine kinase activities resulting in insufficient activation of the PI3K/Akt signaling cascade upon insulin stimulation [47, 48]. Western blot analysis to study the effect of aM1 on PI3K/Akt signaling showed an increased level of phosphorylated Akt signaling cascade proteins, including PDK1 and Akt (Fig. 4B, D). However, in insulin-resistant cells, the compromised INSR-mediated phosphorylation cascade prevents activation of the Akt signaling pathway. Western blot analysis also showed inhibition of Akt signaling cascade activation and that levels of phosphorylated PDK1 and Akt remained unchanged following 100 nM insulin stimulation (Fig. 4B, D). However, aM1 treatment of insulin-resistant myotubes was associated with 1.2- and 1.4-fold increases in phosphorylated PDK1 (Ser242) and Akt (Thr308 and Ser473), respectively, compared to basal levels (Fig. 4B, D). In addition, aM1 treatment produces a similar effect in insulin-resistant adipocytes, with phosphorylation levels of PDK1 (Ser242), Akt (Ser473 and Thr308) increased by 1.4-fold and 1.5-fold, respectively (Fig. S7B, D). Like wild-type myotubes, metformin treatment had no significant effect on PDK1 and Akt phosphorylation in insulin-resistant myotubes, and phosphorylated PDK1 and Akt remained at basal levels after metformin treatment (Fig. 4B, D). Overall, the Western blot analysis revealed that aM1-mediated activation of the Akt signaling cascade occurs independently of insulin receptor-mediated RTK signaling.

INSR activation involves INSR phosphorylation, which was assessed using a membrane-based antibody array. Our array analysis did not detect phosphorylated INSR in aM1-treated cell lysates (Fig. S8), indicating that aM1 might not interact with INSR and which provides support for an insulin receptor-independent mechanism for aM1-mediated Akt-activation.

To corroborate our findings from Western blots and antibody arrays, we investigated aM1-mediated glucose uptake under insulin-inhibited conditions in which cells were treated with an INSR inhibitor, hydroxy-2-naphthalenylmethylphosphonic acid (HNMPA). Insulin-mediated glucose uptake was completely blocked by HNMPA treatment (Fig. 5B). In contrast, aM1-mediated cellular glucose uptake was not affected by HNMPA co-treatment, and a 1.6-fold increase in glucose uptake was seen following aM1 treatment with or without HNMPA (Fig. 5B). Together these findings support the role of Akt signaling in aM1-mediated glucose uptake.

### Transcriptome profiling shows that aM1 modulates gene expression in insulin-resistant C2C12-myotubes

To determine how aM1 alleviates insulin resistance, we profiled the transcriptome of insulin-resistant C2C12 myotubes treated with or without aM1. In control experiments, 12,582 genes (Reads Per Kilo-Base of transcript per Million fragments mapped [RPKM] > 1) were detected using insulin-resistant C2C12 myotubes (Fig. S9A–C and S10A–C). In contrast, aM1-treated cells identified 812 differentially expressed genes (DEGs), with 353 and 459 showing significant up- and down-regulation, respectively (Fig. S10A–C). Quantitative RT-qPCR analysis showed a linear relationship between untreated and aM1-treated cells (Fig. S9A–C). Pathway analysis revealed that upregulated genes are mainly related to metabolism pathways involving phospholipids, glycosaminoglycans, glycosphingolipids, and carbohydrates, as well as transcription (nuclear receptor transcription pathway), signaling (erythropoietin activates phosphoinositide-3-kinase (PI3K) pathway and PI5P, PP2A, and IER3 regulated PI3K/Akt signaling pathway) and cellular transport (VLDLR internalization and degradation pathway, clathrin-mediated endocytosis pathway and SLC-mediated transmembrane transport pathway) processes (Fig. S11A, B). In addition, cholesterol biosynthesis and interferon signaling pathways were among the significantly downregulated pathways in aM1-treated insulin-resistant cells (Fig. S12A, B). Overall, transcriptome analysis demonstrated that aM1 alters the expression of insulin-stimulated signal transduction pathways and related genes in insulin-resistant cells and thus could enhance insulin sensitivity.

### α-astratide aM1 improves insulin sensitivity in insulin-resistant cells

**Fig. 6:**
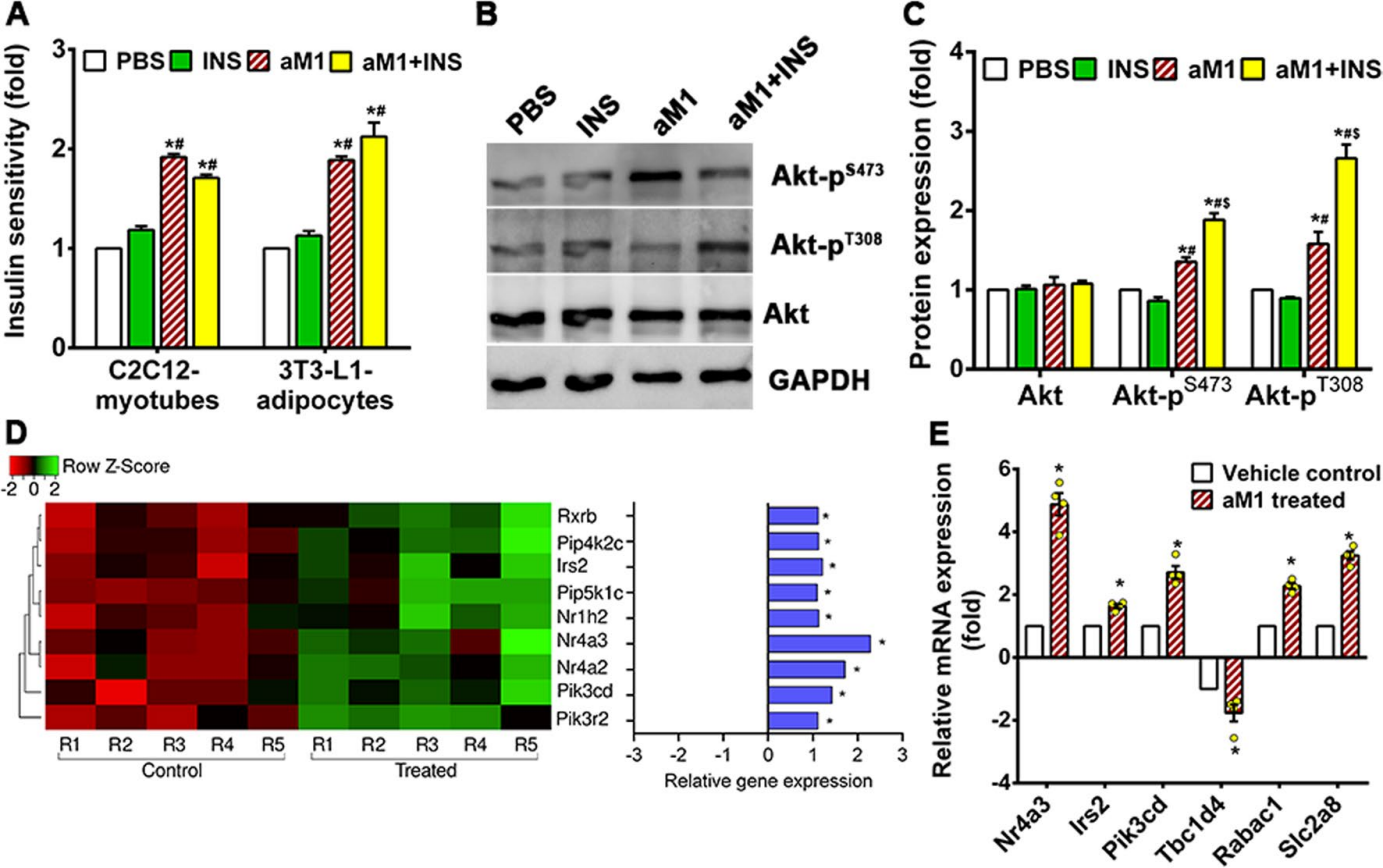
aM1 improves insulin sensitivity in insulin-resistant cells. **A** Relative insulin sensitivity in insulin-resistant (IR) myotubes and adipocytes. After 24 h pretreatment with mock or aM1, the cells were stimulated or not with 100 nM insulin (INS) for 30 min. Glucose uptake of cells after insulin stimulation was quantified using a 2NBDG assay to estimate relative insulin sensitivity, which is expressed as the mean value (± SD) of three independent experimental replicates (analysis of variance (ANOVA) with Sidak's multiple comparisons test). *p < 0.05 compared to PBS control and ^#^*p* < 0.05 compared to INS control. **B** Western blot analysis of Akt phosphorylation in IR myotubes after insulin stimulation with or without aM1 pretreatment. GAPDH served as a loading control. **C** Quantitative analysis of western blot data is depicted as mean ± SD (*n* = 3, ANOVA with Tukey's multiple comparisons test). *p < 0.05 versus PBS control, and ^#^*p* < 0.05 versus INS control and ^$^*p* < 0.05 versus aM1 control. **D** Heat map of differentially expressed genes related to the insulin signaling pathway with opposite profiling compared to aM1-treated-versus-control in IR myotubes, *n* = 5; *p < 0.05 versus control. **E** RT-qPCR analysis of normal proximal insulin signaling genes in IR myotubes. The IR myotubes received mock or aM1 Treatment for 24 h. Gene expression levels in each group were normalized with respect to 18S expression and exhibited a fold-change relative to control groups. Data are expressed as mean ± SEM (*n* = 4, ANOVA with Sidak's multiple comparisons test). *p < 0.05 versus control groups

In T2DM, insulin-sensitive tissues gradually lose insulin sensitivity resulting in insulin resistance and significantly contributing to T2DM complications. In turn, improved insulin sensitivity may improve T2DM management. We evaluated the insulin sensitivity of insulin-resistant C2C12 myotubes and 3T3-L1 adipocytes to investigate this possibility. Cells were treated with antidiabetic agents or aM1, followed by 100 nM insulin stimulation and measurement of glucose uptake using a 2-NBDG-uptake assay to assess insulin sensitivity. Insulin-resistant myotubes had decreased insulin sensitivity and glucose uptake that remains near basal levels after insulin stimulation (Fig. 6A). Cells treated with aM1 or metformin showed 2- and 1.7-fold increases in glucose uptake, respectively, indicating a substantial improvement in insulin sensitivity (Fig. 6A). Insulin-resistant adipocytes also had significantly improved insulin sensitivity following aM1 treatment as evidenced by a 1.9-fold increase in glucose uptake relative to untreated cells (Fig. 6A).

Measurement of phosphorylated Akt levels after aM1 pretreatment in insulin-resistant-C2C12 myotubes in Western blot analysis of insulin signaling pathways showed that insulin stimulation did not activate PI3K-mediated signaling cascades in insulin-resistant cells, and phosphorylated Akt levels remained at basal levels (Fig. 6B, C). In contrast, aM1 pretreatment increased insulin-mediated Akt phosphorylation at Ser473 and Thr308 by 1.9- and 2.6-fold, respectively, in resistant myotubes when compared to vehicle control cells (Fig. 6B, C). Meanwhile, total Akt levels remain unchanged in treated cells (Fig. 6B, C).

Transcriptome analysis showed that PI3K/Akt signaling pathways genes such as Pik3cd, Pik3r2, Pip4k2c, Pip5k1c, and Irs2 are significantly upregulated upon aM1 treatment (Fig. 6D, E), suggesting that aM1-mediated reactivation of PI3K/Akt signaling could enhance insulin sensitivity in insulin-resistant myotubes. Recent studies indicated that orphan nuclear receptors may play a crucial role in metabolism and energy homeostasis [49, 50]. Indeed, overexpression of NR4A nuclear receptors enhances overall insulin activity and improves insulin sensitivity during insulin resistance [49, 50]. We also found that orphan nuclear receptors, including Nr1h2, Nr4a2, and Nr4a3, are significantly upregulated in aM1-treated myotubes (Fig. 6D, E). This result suggests that aM1-mediated induction of PI3K/Akt signaling proteins and orphan nuclear receptors in insulin-resistant myotubes may contribute to the insulin-sensitizing action of aM1.

### aM1 restores lipid homeostasis and limits intracellular lipid accumulation

**Fig. 7:**
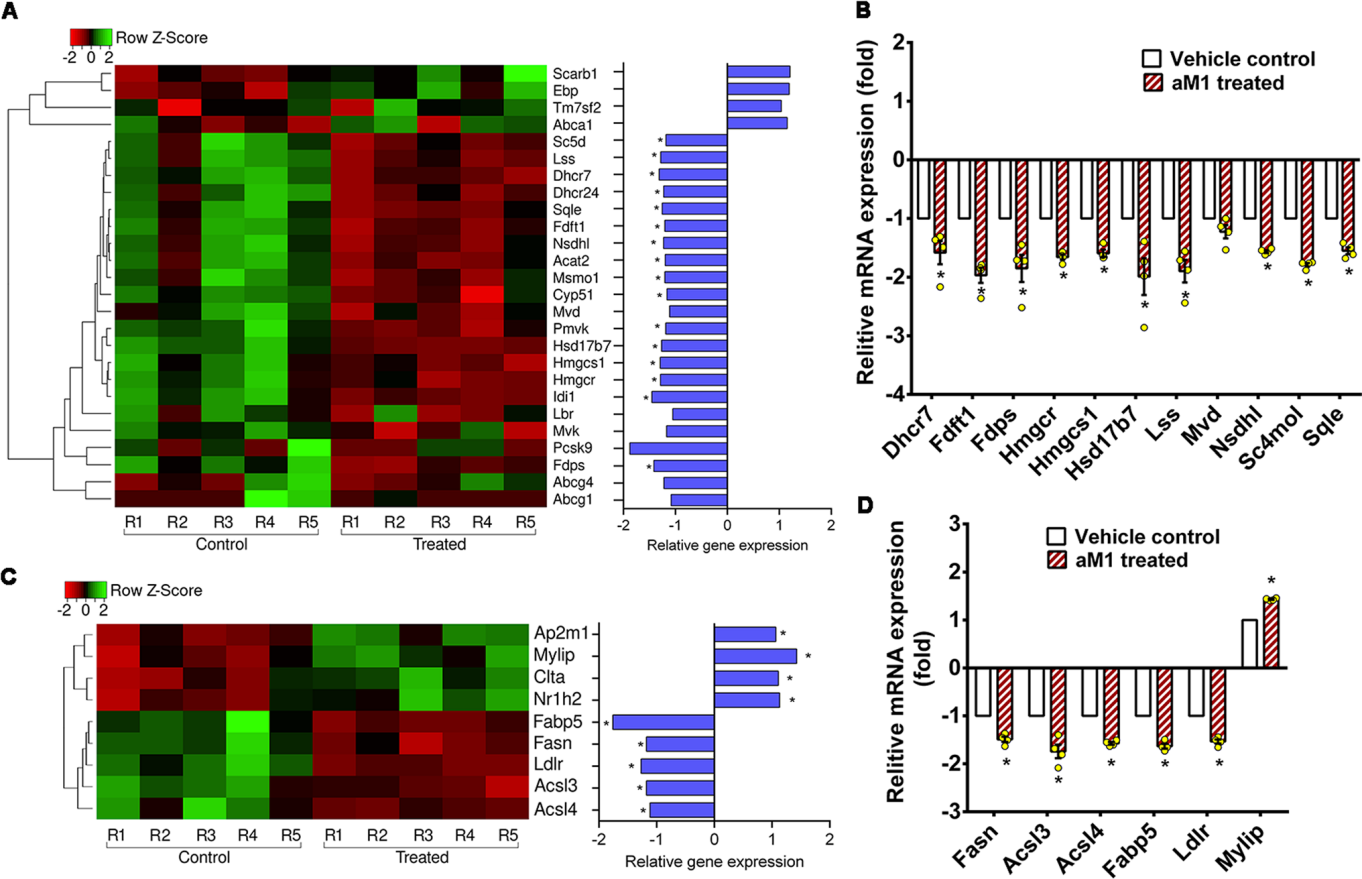
aM1 down-regulates expression of genes related to lipid metabolism in insulin-resistant cells. **A** Heat map of differentially expressed genes related to cholesterol biosynthesis with opposite profiling in a comparison of aM1-treated vs. control in insulin-resistant (IR) myotubes, *n* = 5; *p < 0.05 compared to control. **B** RT-qPCR analysis of genes related to cholesterol biosynthesis in IR myotubes following aM1 treatment. The fold-change in gene expression levels between aM1-treated and control and data normalization were determined with respect to the 18S gene; (*n* = 4, analysis of variance (ANOVA) with Sidak's multiple comparisons test), *p < 0.05 compared to control. **C** Heat map of differentially expressed genes involved in the fatty acid synthesis and uptake with opposite profiling to compare aM1-treated vs. control in IR myotubes, *n* = 5; *p < 0.05 compared to control. **D** RT-qPCR analysis of genes related to cholesterol biosynthesis in IR myotubes after aM1 treatment. The fold-change and data normalization for gene expression levels between aM1-treated and control was done with respect to the 18S gene. Data are presented as mean ± SEM (*n* = 4, ANOVA with Sidak's multiple comparisons test). *p < 0.05 compared to control

**Fig. 8:**
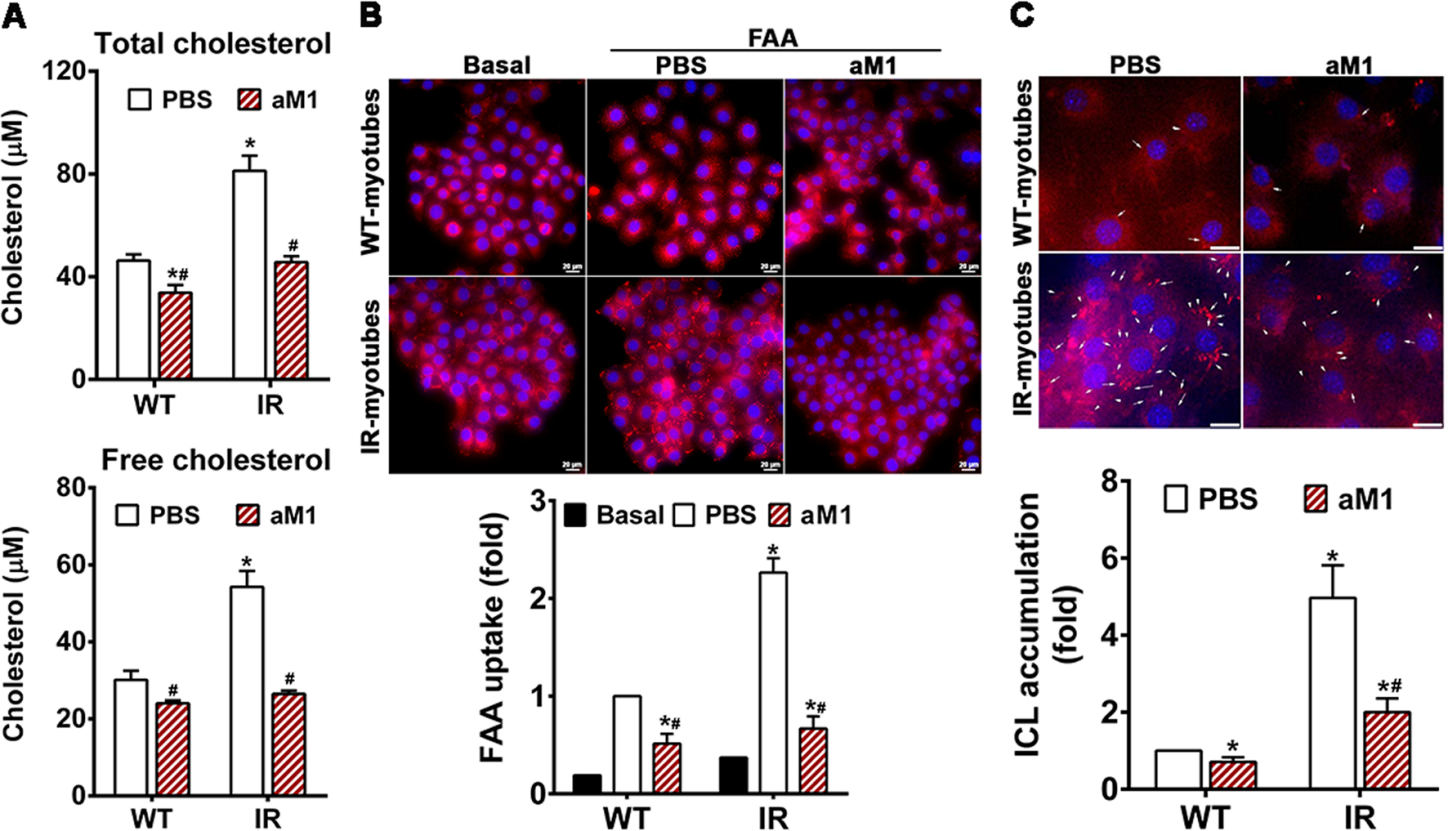
aM1 restores lipid homeostasis and prevents intracellular lipid (ICL) accumulation in insulin-resistant C2C12-myotubes. **A** Intracellular profiling to compare aM1-treated and control in wild type (WT) and insulin-resistant (IR)-C2C12 myotubes. Total intracellular cholesterol (free cholesterol + cholesterol esters) (top) and free cholesterol (bottom) were quantified using commercially available kits, and the data presented are the representative mean ± SD (*n* = 3, analysis of variance (ANOVA) with Tukey's multiple comparisons test) of experimental triplicates. *p < 0.05 compared to WT-PBS control and ^#^*p* < 0.05 compared to IR-PBS control. **B** Free fatty acid (FAA) uptake analysis of C2C12-myotubes. WT and IR-myotubes were pretreated overnight with aM1 and serum-deprived for 6 h, followed by incubation with a 25 µM FAA mixture (2:1 oleic acid and palmitic acid). FAA uptake was visualized through lipid droplet staining with Nile red (red dots), and the nucleus was counterstained with Hoechst 333241 (blue) (scale bar = 20 µm). Relative quantification of FAA uptake was based on average lipid droplet count/cell, and statistical significance was calculated based on 100 cell images from three biological replicates (ANOVA with Tukey's multiple comparisons test); *p < 0.05 compared to WT-PBS control and ^#^*p* < 0.05 compared to IR-PBS control. **C** aM1 reduces ICL accumulation in IR cells. ICL accumulation was assessed after treatment with 20 µM aM1 for 24 h. White arrows indicate lipid droplets stained with Nile red. Nuclei were counterstained with Hoechst 333241 (blue) (scale bar = 20 µm). Relative quantification of ICL was based on the average lipid droplet count/cell, and statistical significance was calculated based on about 100 individual images from three biological replicates (ANOVA with Tukey's multiple comparisons test); *p < 0.05 compared to WT-PBS control and ^#^*p* < 0.05 compared to IR-PBS control

Dysregulated lipid homeostasis impairs the metabolic activities of insulin, leading to insulin resistance in TD2M patients [36–39]. Our transcriptomic analysis revealed the effects of aM1 on lipid homeostasis in insulin-resistant myotubes. Significant downregulation of cholesterol biosynthesis pathway genes, including Acat2, Hmgcs1, Hmgcr, Pmvk, Idi1, Fdps, Fdft1, Sqle, Lss, Cyp51, Msmo1, Nsdhl, Hsd17b7, Sc5d, Dhcr24, and Dhcr7, was observed in aM1-treated resistant myotubes (Fig. 7A and S12B). The RT-qPCR results confirmed differential expression of genes related to cholesterol biosynthesis (Fig. 7B) and showed a highly significant correlation with gene expression levels measured by RNA-seq (Fig. S9C). Intracellular cholesterol quantification to examine the physiological effect of aM1-mediated suppression of cholesterol biosynthesis-related genes showed a twofold increase in intracellular free cholesterol in insulin-resistant C2C12 myotubes compared to the wild-type cells (Fig. 8A). Interestingly, aM1 treatment reduced both total cholesterol and free cholesterol content to basal levels in insulin-resistant myotubes (Fig. 8A). Subsequent estimates of intracellular cholesterol esters confirmed that total cholesterol levels were elevated in insulin-resistant cells. This elevation was absent following aM1 treatment (Fig. 8A).

Both transcriptome and RT-qPCR data suggested that aM1 treatment also suppressed the expression of genes related to fatty acid synthesis, including Fasn, Acsl3, and Acsl4 (Fig. 7B, D). This effect could reduce the synthesis of long-chain fatty acids that, in turn, would prevent intracellular lipid accumulation and lipid-induced insulin resistance in muscle. Treatment with aM1 suppressed the expression of lipid transport-related genes Ldlr and Fabp5 and upregulated Mylip, which degrades Ldlr (Fig. 7B, D), indicating a negative impact of aM1 on cholesterol and lipid influx that would, in turn, reduce intracellular lipid accumulation.

We further examined the lipid-lowering effect of aM1 using Nile red staining to quantify the number of intracellular lipid droplets. Nile red staining showed a threefold increased intracellular lipid accumulation in insulin-resistant cells relative to wild-type cells (Fig. 8C). aM1 treatment reduced this intracellular lipid accumulation by 1.6-fold in insulin-resistant cells compared to vehicle control cells (Fig. 8C). We also measured fatty acid uptake in myotubes and found that uptake of a mixture of oleic acid and palmitic acid (2:1) increased significantly (2.3-fold) when insulin-resistant cells were treated with aM1 when compared to vehicle control treated cells (Fig. 8B).

Our overall findings reveal a lipid-lowering effect of aM1 mediated by the reduction of long-chain fatty uptake and cholesterol production in both wild-type and insulin-resistant cells. The impact of aM1 was more prominent in insulin-resistant cells than in the wild-type counterparts (Fig. 8B, C). Our findings indicate that aM1 substantially influences lipid homeostasis by preventing intracellular lipid accumulation and its pathological effects on insulin activities. Such an effect on lipids may contribute to the insulin-sensitizing action of aM1.

### aM1 mitigates lipid-mediated insulin resistance

**Fig. 9:**
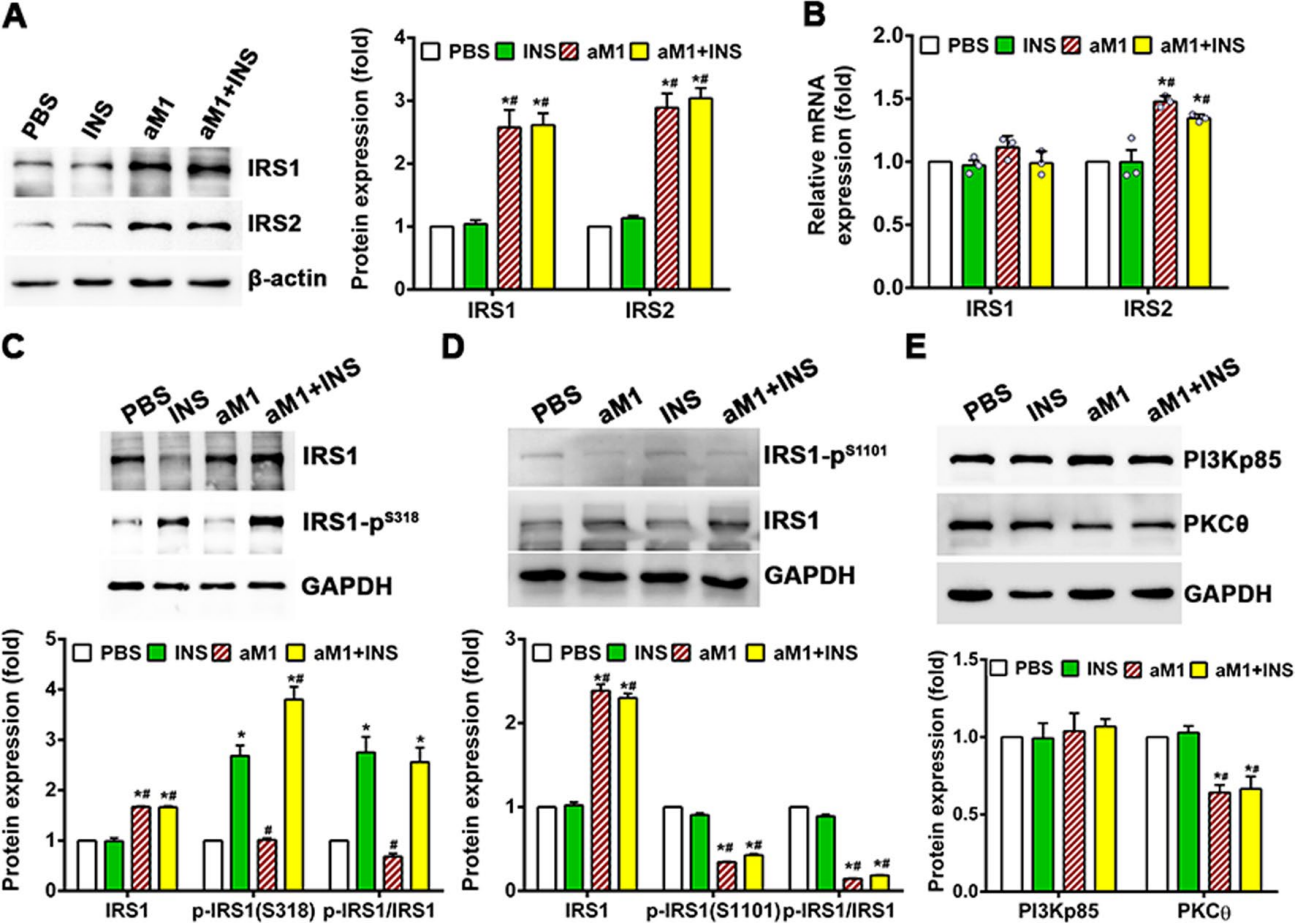
aM1 limits IRS1/2 degradation in insulin-resistant myotubes. **A** Western blots showing the relative protein levels of IRS1/2 in insulin-resistant (IR.) C2C12-myotubes. After 24 h aM1 treatment, the cells were mock-stimulated or treated with insulin (INS) for 30 min. Data shown are representative western blot images and means ± SD (*n* = 3, analysis of variance (ANOVA) with Tukey's multiple comparisons test). *p < 0.05 versus PBS control and ^#^*p* < 0.05 versus INS-treated control groups. **B** IRS1/2 gene expression analyzed by RT-qPCR under respective experimental conditions. Data for each group were normalized and represented the fold change relative to the PBS control group. Data are stated as mean ± SEM (*n* = 4, ANOVA with Tukey's multiple comparisons test). *p < 0.05 versus PBS control and ^#^*p* < 0.05 versus INS-treated group. Western blots of IRS1 at S318 (**C**) and S1101 **D** phosphorylation levels. Quantitative data are mean ± SD (*n* = 3, ANOVA with Tukey's multiple comparisons test). *p < 0.05 versus PBS control and ^#^*p* < 0.05 versus INS-treated control groups. **E** aM1 suppressed PKCθ expression in IR C2C12 myotubes. Data are expressed as mean ± SD (*n* = 3, ANOVA with Tukey's multiple comparisons test). *p < 0.05 versus PBS control and ^#^*p* < 0.05 versus INS-treated control groups

The impact of aM1 on lipid-mediated insulin resistance was assessed by studying PKCθ-mediated IRS1/2 degradation in insulin-resistant C2C12 myotubes using a Western blot-based quantitative analysis of respective pathway proteins. Protein expression analysis showed that aM1 treatment increased the amount of IRS1/2 by 2.5-fold compared to control or insulin-treated insulin-resistant cells (Fig. 9A). In aM1-treated cells, mRNA expression of IRS1 remained unchanged, and IRS2 expression was increased by 1.2-fold (Fig. 9B). In addition, IRS1 phosphorylation at Ser318 increased significantly after insulin treatment of insulin-resistant cells, and aM1 pretreatment further increased phosphorylation levels (Fig. 9C). In comparison, the ratio of phosphorylated IRS1 to total IRS1 was unchanged upon insulin stimulation in both mock- and aM1-pretreated insulin-resistant myotubes (Fig. 9C). Together, these results indicate that aM1 prevents IRS1/2 degradation in insulin-resistant cells and restores IRS1/2 levels that, in turn, improves the insulin sensitivity of insulin-resistant cells. This finding was supported by measuring IRS1 phosphorylation at Ser1101 in insulin-resistant cells with Western blot results confirming a significant reduction in phosphorylated IRS1 (Ser1101) in aM1-treated groups and significant increases in total IRS1 levels (Fig. 9D). The ratio of phosphorylated IRS1 (Ser1101) to total IRS1 was decreased considerably upon aM1 treatment (Fig. 9D), confirming that IRS1 degradation in insulin-resistant cells is inhibited upon aM1 treatment.

To examine IRS1 degradation, we assessed PKCθ expression by Western blot, which showed significant (1.5-fold) down-regulation of PKCθ expression in aM1-treated insulin-resistant cells (Fig. 9E). This blotting data suggest that aM1 inhibits lipid-mediated induction of the PKCθ and prevents activation of the DAG-PKCθ axis and degradation of IRS1/2 and in turn, restores insulin sensitivity in insulin-resistant cells.

## Discussion

In this study, we report that α-astratide aM1 isolated from huáng qí, a popular medicinal herb widely prescribed for treating diabetes [21–26], is principally involved in its antidiabetic activity. We show that the highly stable and disulfide-dense aM1 is a cell-penetrating microprotein mimicking insulin to improve cellular glucose uptake for both normal and insulin-resistant cells. Additionally, aM1 modulates lipid metabolism genes and exerts lipid-lowering effects to alleviate lipid-induced insulin resistance. The combined glucose- and lipid-lowering properties of aM1 offer new opportunities and promising prospects for developing new options in diabetes management.

In agreement with previous reports that leginsulins affect glucose metabolism [22–24], we showed that aM1, which shares high sequence and structure similarity to leginsulin, also affects glucose metabolism. In vitro, aM1 treatment resulted in a burst of glucose uptake, which can be observed in multiple cell types, indicating that aM1 can mediate improvements in peripheral and hepatic glucose uptake. Glucose uptake in peripheral tissues is primarily channeled through the insulin-sensitive GLUT4 glucose transporter [31–34]. Insulin resistance impairs the blood-glucose-lowering effect of circulating or injected insulin [44–46]. We showed that aM1 treatment significantly improves glucose uptake in insulin-resistant cells, comparable to the widely used antidiabetic therapeutics, such as metformin and pioglitazone. In addition, aM1 also restored insulin sensitivity to insulin-resistant cells.

**Fig. 10:**
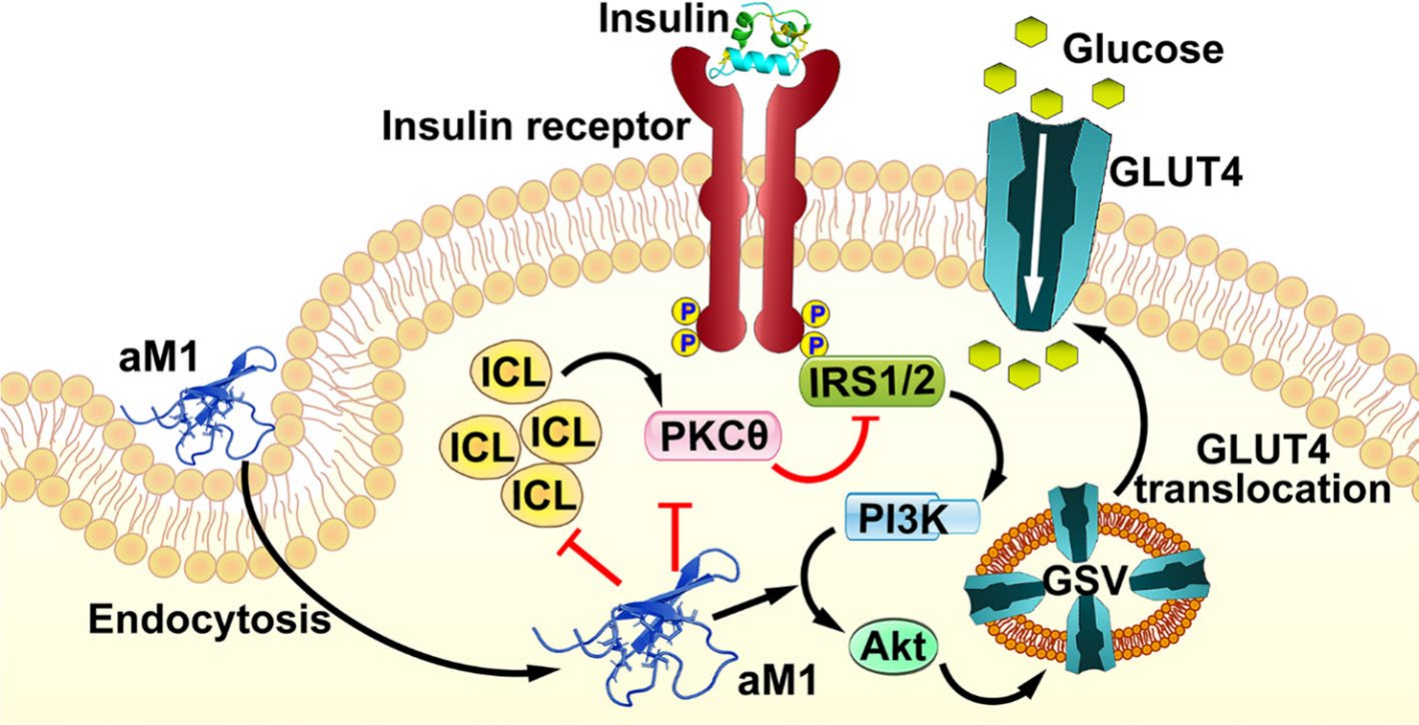
Schematic representation of molecular events in aM1-mediated glucose uptake by insulin-resistant cells. Insulin binding to the insulin receptor activates insulin receptor kinase, which in turn activates IRS1/2, PI3K, and Akt. Activated Akt further triggers GLUT4 translocation to the plasma membrane and initiates glucose internalization. In insulin-resistant cells, accumulation of intracellular lipid (ICL) activates the DAG-PKCθ axis and impairs insulin signaling by degrading IRS1/2 and PI3K/Akt pathway proteins. aM1 enters cells through endocytosis, activates the PI3K/Akt pathway, and promotes glucose uptake. In insulin-resistant cells, aM1 inhibits ICL accumulation by restoring lipid homeostasis, further blocking activation of the DAG-PKCθ axis. Finally, aM1 prevents IRS degradation and restores insulin signaling. *IRS1/2* insulin receptor substrate 1/2, *DAG* diacylglycerol, *PKCθ*: protein kinase C-theta, *PI3K* phosphoinositide 3-kinase, *Akt* protein kinase B, *ICL* intracellular lipid, *GLUT4* glucose transporter type 4

How does aM1 exert its insulin-mimicking functions? Glucose uptake is controlled through insulin-dependent and insulin-independent signaling pathways, and in both of these signaling, PI3K/Akt signal transduction plays a central role [42–44]. Activating the PI3K/Akt pathway augments glucose transport and facilitates cellular glucose uptake to lower blood glucose levels [42–44]. We showed that aM1 enters cells by endocytosis and activates the Akt pathway directly. The following results support our conclusion: (1) aM1 treatment increased PDK1 phosphorylated at Ser241, resulting in Akt phosphorylation at Thr308 and Ser473 sites and Akt activation [42–44, 51]; (2) inhibition of Akt is accompanied by a reduction of aM1-mediated glucose uptake; (3) comparable effect of aM1 treatment are found in both wild-type and insulin-resistant cells; (4) aM1 does not significantly impact insulin receptor auto-phosphorylation [45, 52]; (5) Inhibiting the insulin receptor has no significant effect on aM1-mediated glucose uptake (Fig. 10). Furthermore, our findings on aM1-mediated Akt activation are strengthened by the activation of the Akt pathway observed with similar legume peptides such as aglycin (from soy) and vglycin (from pea) [53]. Furthermore, aM1 upregulates the expression of normal proximal insulin signaling genes, including IRS2, in insulin-resistant C2C12-myotubes. This upregulation could contribute to restoring insulin sensitivity [54]. Upregulated expression of the orphan nuclear receptor NR4A in response to aM1 treatment may also enhance insulin activity and restore insulin sensitivity of insulin-resistant cells [49, 50]. The unusual and indirect insulinotropic effect of aM1 to mediate glucose uptake may involve other factors such as Ca^2+^ ions, which also play a role in glucose uptake [55] and which would require further studies. However, the significance of the cell-penetrating property of the 37-residue aM1 peptide cannot be understated because it plays a central role in facilitating the entry of aM1 into cells and targeting the cellular pathways activated by the insulin receptor, effectively mimicking insulin activities.

A significant finding of this report is that aM1 mitigates insulin resistance. In insulin resistance, the normal insulin-mediated activation of PI3K/Akt signaling is impaired, disrupting the translocation of GLUT4 and glucose uptake. [47, 48]. Recent reports revealed that intracellular lipid accumulation promotes pathological responses that eventually inactivate insulin signaling [56–58]. The relationships between insulin resistance and intracellular lipid accumulation are complex and vary according to physiological and environmental factors [56–58]. Thus far, mechanisms associated with lipid-induced insulin resistance in ectopic tissues are unclear. Increased intracellular cholesterol levels significantly contribute to lipid-induced insulin resistance in ectopic tissues, including skeletal muscle [59–61]. In this study, we observed an increase in intracellular cholesterol accumulation and fatty acid uptake in insulin-resistant myotubes, leading to excessive accumulation of intracellular lipid droplets. The accumulated lipids can trigger lipid-mediated activation of the DAG-PKCθ axis in insulin-resistant cells [56–58], which dissociates IRS1/2 from the insulin receptor complex and phosphorylates IRS1 at Ser1101 marks them for degradation linked with insulin insensitivity [62, 63]. While aM1 treatment alters the expression of lipid synthesis and lipid uptake, genes including cholesterol and long-chain fatty acid synthesis and uptake-related genes restore lipid homeostasis in insulin-resistant myotubes by lowering lipid synthesis and uptake, eventually inhibiting intracellular lipid accumulation. Moreover, inhibition of lipid accumulation further blocks PKCθ-mediated IRS1/2 degradation and ameliorates PI3K/Akt signaling disruptions to enhance overall insulin in insulin-resistant cells (Fig. 10).

The identification of antidiabetic aM1 is promising because a recent trend in antidiabetic drug development is shifting from small molecules to peptides, exemplified by glucagon-like peptides. This shift is partly due to their advantages of high on-target specificity and low off-target side effects [64, 65]. However, peptide-based drugs also have limitations. They include their short half-lives and the need for injectable administration [64–68]. Orally active peptides could address some of these limitations. In agreement with previous reports [13–17, 20, 34], the disulfide-knotted aM1 is structurally compact and resistant to thermal, chemical, and enzymatic degradation. Moreover, the hydrophobic nature and a 4 kDa MW of aM1 enable it to penetrate cells to target intracellular protein–protein interactions, likely more efficiently than small-molecule drugs. Interestingly, the disulfide arrangement and structural features of aM1 share certain similarities to the linear forms of the cyclotide family, exemplified by hedyotide B2 from *Hedyotis biflora* [69]. Our earlier studies have demonstrated a successful strategy to transform proteolytically labile peptides, such as bradykinin inhibitors, into proteolytic-resistant and orally active analogs, by molecular grafting a labile bradykinin peptide onto a cyclotide framework [70]. This example provides a precedent to develop plant-derived highly disulfided peptides for oral administration. Furthermore, we have recently reported that such cystine-dense peptides with MW 2–4 kDa appeared much more common than previously thought [12, 14, 15, 18, 19]. Noteworthy examples include roseltides [15, 18, 19], β-ginkgotides [14], and ginsentides [12, 17], which exhibit diverse bioactivities with potential therapeutic applications. These cell-penetrating peptides offer certain advantages in targeting intracellular proteins and the potential for oral administration.

Huáng qí has been used for centuries as food and herbal medicine and is generally considered safe. Our studies also showed that aM1 is nontoxic to mammalian cells [20]. As a plant-derived biologic, aM1 can be produced using plants as bioreactors, capitalizing on their bioprocessing capabilities [71–73]. Overall, the "first-in-class" plant-derived insulin mimetic aM1 shows high promise as a lead and approach for developing a new class of drugs for diabetes. However, further in vivo animal studies are necessary to validate its therapeutic potential.

## Methods

### Reagents

Unless otherwise indicated, all chemicals and reagents were from Sigma-Aldrich, USA. Antibodies against β-actin (C4), GAPDH (6C5), Glut4 (IF8), Myogenin (5FD), and Alexa fluor 488 conjugated c-Myc (9E10) were from Santa Cruz Biotechnology, Santa Cruz, USA. Anti-Akt (pan) (C67E7), anti-phospho-Akt (Ser473) (D9E), anti-phospho-Akt (Thr308) (C31E5E), anti-IRS-1 (D23G12), anti-phospho-IRS-1 (Ser1101), anti-phospho-IRS-1 (Ser307), anti-phospho-IRS-1 (Ser318) (D51C3), anti-phospho-IRS-1 (Ser612) (C15H5), anti-IRS-2 antibody, anti-phospho-PDK1 (Ser241) (C49H2), HRP-linked anti-mouse IgG, and HRP-linked anti-rabbit IgG antibodies were obtained from Cell Signalling Technology, USA. Clarity Max™ Western ECL blotting substrates (1705062) were purchased from Bio-Rad Laboratories, Italy. The cell line C2C12 (ATCC CRL-1772), 3T3-L1 (ATCC CL-173), and HEPG2 (ATCC HB-8065) were acquired from ATCC (VA, USA).

### Extraction and purification of α-astratide aM1

Peptide extraction was performed as described by Tam et al*.* [13] and Huang et al*.* [20] with minor modifications. In brief, dried huáng qí (*Astragalus membranaceus*) roots were pulverized and blended with deionized water (100 g/l). The extract was clarified by centrifugation and filtration through a 0.22-μm membrane (Thermo Fisher Scientific, USA). Solid-phase peptide extraction using DAVISIL C18 Silica Resin (Grace, USA) was used to isolate peptides. Purification was performed using multiple rounds of Reverse-Phase High-Performance Liquid Chromatography (RP-HPLC) with a C18 column (250 mm × 22 mm, 5 μm particle size) (Phenomenex, USA) and a linear gradient of 1%/min of 10–80% buffer B (0.1% TFA in ACN) and buffer A (0.1% TFA in HPLC water) at a flow rate of 5 ml/min. Analytical RP-HPLC with a C18 column (250 mm × 4.6 mm, 5 μm particle size; Phenomenex, USA) was carried out with a flow rate of 1 ml/min, and the same gradient was used for final purification. Peptide identification was performed using MALDI-TOF MS. Presence of native aM1 was further confirmed with RP-HPLC analysis of a 1:1 mixture of reference aM1 (our previously isolated aM1 by Huang et al*.* [20]) and isolated aM1 (presently isolated). We yielded 20 mg of purified peptide with ~ 95% purity form 1 kg of dried roots.

### Site-specific N-terminal fluorescent labeling of α-astratide aM1

AF488 NHS ester (Lumiprobe, USA) was used to label the N terminus of purified α-astratide aM1. The labeling reaction was carried out overnight at room temperature in 100 mM sodium bicarbonate buffer (pH 8). Labeled aM1, AF488-aM1 was purified and analyzed using RP-HPLC and MALDI-TOF MS (Fig. S5). The purity of labeled aM1 was calculated from the HPLC chromatogram and achieved ~ 96% purity.

### Cell culture and treatment

Muscle myoblast cells (C2C12), embryonic mouse fibroblast cells (3T3-L1), and human hepatocellular carcinoma cells (HEPG2) were maintained in complete growth medium (DMEM supplemented with 10% FBS and antibiotics (Penicillin 100 IU/ml and Streptomycin 100 µg/ml)) at 37 °C, 5% CO_2_ for further use.

### C2C12 myoblast and 3T3-L1 fibroblast differentiation and insulin-resistant phenotype

Differentiation of C2C12 cells was achieved by replacing the complete growth medium with differentiation media, MF (equal mixture of two serum-free media (MCDB201 and Nutrient Mixture F-12 Ham medium), and 0.05% BSA), and culture for three days. The medium was replaced every 12 h, and differentiation was confirmed by myogenin expression (Fig. S13A, B). An insulin-resistant phenotype was produced by differentiating the cells in differentiation media (MFI) containing 100 nM insulin.

3T3-L1 cells were maintained in growth medium (high-glucose DMEM [Dulbecco's modified Eagle's medium; Gibco], 10% FBS (fetal bovine serum; Gibco) and 1% penicillin/streptomycin (Gibco)]). Confluent 3T3-L1 fibroblast cells were then incubated with MDI induction medium (1 μM dexamethasone, 0.5 mM IBMX (isobutylmethylxanthine), and 100 nM insulin in high-glucose DMEM containing 10% FBS. At 2 days post-confluence and 2 days after exposure to the MDI induction medium, the medium was replaced with 100 nM insulin containing high-glucose DMEM supplemented with 10% FBS. Thereafter, the medium was replaced with a growth medium every 2 days to maintain the cells in culture. Cells were maintained in a 100 nM insulin-containing growth medium for the insulin-resistant phenotype. Differentiation was confirmed with Oil Red to visualize lipid droplets (Fig. S13C).

### Drug treatment

Cell Culture and Treatments: Cells were maintained in their respective growth media. Pre-culture cells were either mock-treated with PBS or treated with vehicle control. The dose–response curve was established using a 0-60 µM dose of aM1 for 24 h. The rest of the experiments were conducted using 20 µM aM1 over a 24 h time period. Positive control cells were treated with 100 nM insulin for 30 min. To investigate the effect of aM1 on glucose uptake, cells were treated with the glucose uptake inhibitor phloretin (100 µM) for 1 h as a negative control. Positive or reference controls for glucose uptake and pathway analysis studies involved cells treated with 20 µM metformin or pioglitazone for 24 h.

Akt activation-mediated signaling was inhibited using the Akt-inhibitor GSK690693. Hydroxy-2-naphthalenylmethylphosphonic acid (HNMPA) was used to block insulin receptor activation. Cells were pretreated with 20 µM aM1 for 24 h and then incubated with 2.5 µM GSK690693 or 100 µM HNMPA for 1 h before cells were stimulated with 100 nM insulin for 30 min. PBS was used for mock-treated groups. All experiments had at least three independent replicates.

### Flow cytometry-based cellular uptake analyses

To examine AF488-aM1 cellular uptake, cells were cultured and incubated with 1 μM AF488-aM1 in serum-free growth medium at 37 °C for 1 h before harvesting and collection by centrifugation at 500×*g* for 5 min. Extracellular fluorescence of the cells was then quenched with 150 μg/ml trypan blue and analyzed using an LSRFortessa X-20 analyzer flow cytometer (Becton Dickinson, USA). Data analysis was performed using 10,000 cells for each experimental condition. Temperature-dependent uptake was evaluated by pre-incubating cells at 4 °C for 30 min and then incubating with AF488-aM1 for 1 h at 4 °C. To assess cellular uptake of AF488-aM1, cells were treated with endocytosis inhibitors, including 50 μM dynasore, 50 μM EIPA (5-(*N*-ethyl-*N*-isopropyl) amiloride), or 50 μg/ml nystatin for 30 min before incubation with AF488-aM1 for 1 h at 37 °C. The cells without AF488-aM1 (blank) were used to examine the background florescent intensity. Experiments were conducted in triplicate.

### Confocal microscopy analysis

To examine the intracellular distribution of AF488-aM1, cells were pre-grown on an 8-well chamber slide (Ibidi, Germany) before incubating with 1 μM AF488-aM1 at 37 °C for 1 h and counterstaining with Hoechst 333241. The media was then replaced after gently washing with PBS. Slides were observed and imaged using a Zeiss LSM 710 confocal microscope.

### 2-NBDG assay to measure glucose uptake

Glucose uptake was measured using 2-NBDG, a fluorescently labeled deoxyglucose analog. Pre-cultured cells were washed twice with serum- and glucose-free DMEM before incubation in serum- and glucose-free DMEM containing 50 µM 2-NBDG for 30 min at 37 °C, followed by washing with ice-cold PBS. The 2-NBDG that was taken up by the cells was extracted by lysing cells in lysis buffer [1% Nonidet P-40, 1% sodium deoxycholate, 40 mM KCl, and 20 mM Tris (pH 7.4)]. Fluorometric quantification was performed using a microplate reader (Tecan Magellan™, Switzerland) with excitation and emission wavelengths of 467 nm and 542 nm, respectively. Protein content was measured with a BCA protein assay to normalize fluorometric results. For microscopy, after incubation with 2-NBDG, the cells were washed with ice-cold PBS and placed in Hank's balanced salt solution (HBSS). Images were captured using the green fluorescence channel of a Nikon ECLIPSE Ti-S inverted microscope system (Nikon, Kanagawa, Japan). Assays were performed in triplicate.

### Glucose consumption assay

Glucose consumption was estimated by measuring the depletion of glucose levels in the growth medium over time. Glucose quantification was carried out using a glucose assay kit (Sigma, USA). Briefly, pre-plated cells were serum-starved overnight and then glucose-starved for 1 h before incubation in low glucose (1 g/l) medium with or without the indicated compounds, including 100 nM insulin and 20 µM aM1. aM1-treated group was pretreated with 20 µM of aM1 for 24 h. After glucose introduction, glucose levels in the culture medium at 0 and 60 min were estimated colorimetrically at an absorbance wavelength of 570 nm. The protein content of each well was measured with a BCA protein assay to normalize glucose uptake results. Statistical significance was calculated based on three individual assays.

### LDH assay

LDH release-based cytotoxicity assays were carried out to evaluate the cytotoxic effects of aM1. In brief, cells were cultured with or without 100 µM aM1 for 24 h. DMSO was used as the vehicle control. Triton X-100 (1%) was used as a positive control for cytotoxic and membrane-damaging effects. The culture medium was collected, and the LDH concentration was determined using a CytoSelect™ LDH Cytotoxicity Assay Kit (CBA-241, Cell Biolabs, Inc., CA.). Quantification was determined calorimetrically at 450 nm. The experiment was conducted in triplicate, and GraphPad Prism (Version 6.01) was used for statistical calculations and bar graphs.

### MTT assay

Cell viability in the presence/absence of aM1 was evaluated using an MTT assay. Cells were pretreated with 100 µM aM1 for 24 h before the MTT reagent was added (final concentration 0.5 mg/ml) and incubated for 2 h at 37 °C. The MTT-containing culture medium was aspirated, and the formazan crystals were dissolved in DMSO for colorimetric quantification at 570 nm using a microplate reader (Tecan Magellan™, Switzerland) with a reference wavelength of 630 nm. DMSO was used as vehicle control, and 1% Triton X-100 was used as a cytotoxicity-inducing agent (positive control). Experimental triplicates were used for statistical analyses.

### GLUT4 translocation assay

The GLUT4 translocation assay was adapted from a published protocol [37]. The pEGFP-myc-GLUT4-mCherry recombinant construct for GLUT4 fusion protein was generated using the pLenti-myc-GLUT4-mCherry plasmid [37] (Addgene plasmid #64049). C2C12 cells were transfected with the recombinant plasmid using a Lipofectamine 3000 Transfection Kit (Thermo Scientific, USA). Stably transfected cells were selected and grown for further assays. C2C12 cells expressing the GLUT4 fusion protein were grown on coverslips in serum-free DMEM medium and treated with 20 μM aM1 for 24 h. The positive control group was treated with 100 nM insulin for 30 min, and the mock control was treated with PBS. After treatment, the culture medium was aspirated, and the cells were washed three times with PBS. Nonpermeabilized cells were immediately fixed with 10% formalin for 30 min, washed, and blocked with 1% BSA for 1 h at RT. After blocking, the cells were incubated with Alexa Fluor 488-conjugated c-Myc antibodies overnight at 4 °C. Subsequently, coverslips were retrieved, mounted onto slides, and visualized using a Nikon ECLIPSE Ti-S inverted microscope.

### Western blot analysis

Each protein sample (30 µg) was resolved on a 10% acrylamide gel and transferred onto a PVDF membrane. Immunoblotting was performed using anti-protein antibodies and detected with an ECL system.

### Insulin sensitivity assay

Insulin-resistant C2C12-myotubes and 3T3-L1-adipocytes were cultured with or without 20 µM aM1 for 24 h. The aM1-pretreated cells were then subjected to stimulation with mock (PBS) or 100 nM insulin for 30 min. Then, the cells were washed twice with serum- and glucose-free DMEM and incubated with 50 µM 2-NBDG for 30 min at 37 °C. The fluorescence of 2-NBDG was measured as described above. Experiments were performed in triplicate.

### Insulin sensitivity assay

Insulin-resistant C2C12-myotubes and 3T3-L1-adipocytes were cultured with or without 20 µM aM1 for 24 h. The aM1-pretreated cells were then subjected to stimulation with mock (PBS) or 100 nM insulin for 30 min. Then, the cells were washed twice with serum- and glucose-free DMEM and incubated with 50 µM 2-NBDG for 30 min at 37 °C. The fluorescence of 2-NBDG was measured as described above. Experiments were performed in triplicate.

### Insulin sensitivity assay

Insulin-resistant C2C12-myotubes and 3T3-L1-adipocytes were cultured with or without 20 µM aM1 for 24 h. The aM1-pretreated cells were then subjected to stimulation with mock (PBS) or 100 nM insulin for 30 min. Then, the cells were washed twice with serum- and glucose-free DMEM and incubated with 50 µM 2-NBDG for 30 min at 37 °C. The fluorescence of 2-NBDG was measured as described above. Experiments were performed in triplicate.

### Insulin sensitivity assay

Insulin-resistant C2C12-myotubes and 3T3-L1-adipocytes were cultured with or without 20 µM aM1 for 24 h. The aM1-pretreated cells were then subjected to stimulation with mock (PBS) or 100 nM insulin for 30 min. Then, the cells were washed twice with serum- and glucose-free DMEM and incubated with 50 µM 2-NBDG for 30 min at 37 °C. The fluorescence of 2-NBDG was measured as described above. Experiments were performed in triplicate.

### RNA-Seq: read processing and DEG analysis

Raw Reads in a FASTQ format were first processed with fastp to obtain clean reads. In this process, all low-quality reads along with reads containing adapter and poly-N sequences were removed from raw data. Subsequently, the Q20, Q30, and GC content of the clean reads was calculated. These high-quality clean reads were used in all downstream analyses.

Data analysis was carried out using publicly available reference genome and gene model annotation data from genome databases (NCBI/UCSC/Ensembl). The paired-end clean reads were aligned to the reference genome using Spliced Transcripts Alignment to a Reference (STAR) software. The RNA-seq alignment algorithm with sequential maximum mappable seed search in uncompressed suffix arrays followed by seed clustering and stitching procedure was applied for alignment. Counting the read number of each mapped gene was done using FeatureCounts. Reads Per Kilobase of exon model per Million mapped reads (RPKM) for each gene were calculated using the gene and reads count map of the respective gene [74].

A DESeq2 R package-based DESeq2 analysis was executed for differential expression analysis to compare two conditions/groups (five biological replicates per condition). The resulting P values were adjusted using Benjamini–Hochberg's method for controlling the false discovery rate (FDR). Genes with an adjusted *P* value < 0.05 found by DESeq2 were categorized as differentially expressed. We also considered the edgeR technique for differential gene expression analysis of two conditions. The read counts were adjusted according to the trimmed Mean of M-values (TMM) for each sequenced library through one scaling normalized factor before differential gene expression analysis. The *P*-values were adjusted using Benjamini–Hochberg's methods. A corrected *P* value of < 0.05 and |log_2_^(Fold Change)^| of 1 were set as the threshold for significantly differential expression.

### Enrichment analysis of DEGs

Bioinformatic analyses, including enrichment analysis of DEGs (Differential Expression Genes), were conducted to understand their relevance to the biological context. R package clusterProfiler was used to test the statistical enrichment of DEGs in KEGG pathways. An adjusted *P*-value > 0.05 indicated significant enrichment for KEGG terms. Reactome enrichment analysis of DEGs with cluster profile is based on an R package that uses biological term classification and enrichment analysis for gene cluster comparison. Reactome terms with adjusted *P*-value > 0.05 were considered to indicate significant enrichment.

### Quantitative reverse transcription-polymerase chain reaction (qRT-PCR)

RNA was extracted using a PureLink RNA mini kit (Invitrogen, USA), and first-strand cDNA synthesis was performed using RevertAid H Minus First Strand cDNA Synthesis Kits (Thermo Fisher Scientific, USA). The primer sequences used are listed in Supplementary Table S1. Real-time PCR amplification was done using an SYBR Green Supermix (Bio-Rad, USA) with a CFX Connect™ Real-Time PCR Detection System (Bio-Rad Laboratories, Singapore). Cycling conditions were enzyme activation at 95 °C for 3 min, denaturation at 95 °C for 10 s, and annealing and extension at 60 °C for 30 s. Relative gene expression was determined from experimental triplicates, and 18S ribosomal RNA expression was used for expression normalization.

### Nile red staining of intracellular lipid droplets

Nile red staining was used to detect intracellular lipid droplets. In brief, pre-grown cells on an 8-well chamber slide (Ibidi, Germany) were incubated with or without aM1 for 24 h. Following treatment, cells were washed with HHBS, stained with 1 μM Nile red for 10 min, and counterstained with 1 μM with Hoechst 333241 for 10 min. Post-stained cells were washed and placed in pre-warmed HHBS and imaged using a Nikon ECLIPSE Ti-S inverted microscope (Nikon, Kanagawa, Japan). Fluorescence intensity and lipid droplet count were quantified using ImageJ Version 1.50b (NIH, USA). The experiment was performed in triplicate, and more than 50 individual cell images for each condition were considered for analysis.

### Free fatty acid uptake assay

A Nile red staining approach was used to detect intracellular lipid droplet accumulation after free fatty acid treatment. In brief, cells grown on an 8-well chamber slide (Ibidi, Germany) were pretreated with or without aM1 for 6 h in a serum-deprived environment. Following serum depletion and pretreatment, cells were incubated overnight in a growth medium containing free fatty acid (25 µM FAA mixture (2:1 oleic acid and palmitic acid)) with or without aM1. Treated cells in a medium without fatty acid were used as a basal control. Intracellular lipid droplets of the post-treatment cells were stained with 1 μM Nile red for 10 min, and the nuclei were counterstained with 1 μM Hoechst 333241. Stained cells were imaged using a Nikon ECLIPSE Ti-S inverted microscope system (Nikon, Kanagawa, Japan). Fluorescence intensity and lipid droplet count were quantified using ImageJ Version 1.50b (NIH, USA). Experiments were performed in triplicate, and more than 50 individual cell images for each condition were considered for the analysis.

### Cholesterol assay

Intracellular cholesterol was quantified using a Cholesterol/Cholesterol Ester-Glo™ Assay kit (Promega, USA). C2C12 cells were grown and differentiated for 3 days on a 48-well plate. After differentiation, cells were treated with aM1 for 24 h. Free cholesterol and cholesterol esters were quantified with a Cholesterol/cholesterol ester-glo™ assay performed in triplicate.

### Statistical analyses

Statistical comparisons were executed with GraphPad Version 6.01 software (USA) using the data from triplicate experiments or as maintained in the respective experiments. One-way analysis of variance (ANOVA) or two-way ANOVA with Tukey's multiple comparisons test or Šidák's multiple comparisons test method was used accordingly for data analysis. At the same time, we maintained the method used in the respective figures. Data are shown as the mean ± standard deviation (SD) or standard error of the mean (SEM), and *p* < 0.05 was considered statistically significant.

### Supplementary Information

Below is the link to the electronic supplementary material.

Supplementary file1 (PDF 4086 KB)

The figures. Fig. S1A–C, S2, S3, S4A–F, S5A, B, S6A–D, S7A–D, S8A–C, S9A–D, S10A, B, S11A, B, S12A–C, and S13 and full blot images of Fig. 4A, B, 6B, 9A, C–E, S6C and S7A, B are provided in Supplementary Data 1 (PDF). The complete DEG list of RNA-seq data and primer list used in RT-qPCR analysis are included in Supplementary Data 2 (Excel files) (XLSX 11452 KB)

## Data Availability

The datasets generated during and/or analyzed during the current study are available in the NCBI Sequence Read Archive (SRA) and can be accessed from SRA using the accession code PRJNA975615.
