# Supplementary material for: Plant-derived cell-penetrating microprotein α-astratide aM1 targets Akt signaling and alleviates insulin resistance
Source: Cell Mol Life Sci. 2023 Sep 16;80(10):293. doi: 10.1007/s00018-023-04937-y (PMC10505102; doi:10.1007/s00018-023-04937-y)
Supplement: Supplementary file 1 — Supplementary file1 (PDF 4086 KB) [file 18_2023_4937_MOESM1_ESM.pdf]

Plant-derived cell-penetrating microprotein α-astratide aM1 targets Akt signaling and alleviates insulin resistance

Bamaprasad Dutta[1], Shining Loo[1, 2], Antony Kam[1, 3], and James P. Tam[1]*

[1]School of Biological Sciences, Nanyang Technological University, 60 Nanyang Drive, Singapore 637551
[2]Academy of Pharmacy, Xi'an Jiaotong-Liverpool University, Suzhou, 215123, China.
[3]Department of Biological Sciences, Xi'an Jiaotong-Liverpool University, Suzhou 215123, China

Running title: Insulin-mimetic microprotein aM1 overcomes insulin resistance

***Correspondence:**
James P Tam
Professor
School of Biological Sciences
Synthetic Enzymes and Natural Products Center
Nanyang Technological University,
60 Nanyang drive, Singapore 637551
Tel: (+65) 6316-2833
Email: jptam@ntu.edu.sg

Supplementary Figures

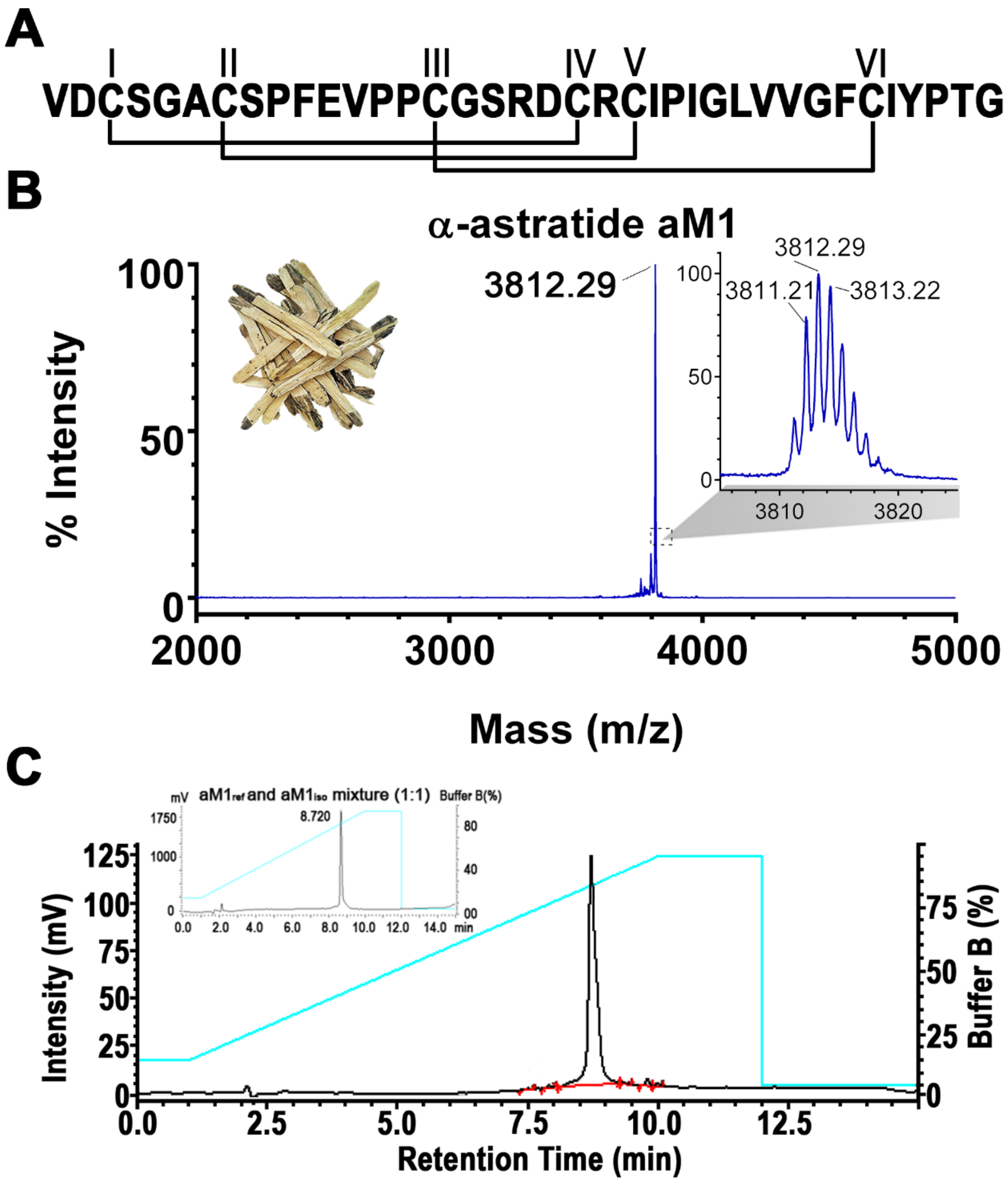

Figure S1: Analytical profiling of α-astratide aM1, after extracted from the dried *A. membranaceus* roots and purified by RP-HPLC. A) Primary amino acid sequence and disulfide connectivity of aM1. B) MALDI-TOF MS analysis of aM1. The calculated mass of aM1 is 3813.43Da, and the observed mass of purified aM1 is 3812.29Da, (see insert) C) HPLC analysis of purified aM1. The integrated peak areas are highlighted with red arrows. The purity of aM1 peptide (~95%) was calculated based on the percentage of the pure peptide of the whole integrated peak area. Insert chromatogram represents the HPLC analysis of a 1:1 mixture of reference aM1 ($aM1_{ref}$) and isolated aM1 ($aM1_{iso}$), shows a single aM1 peak at a retention time of 8.7min, confirming the presence of native aM1 in the isolated sample.

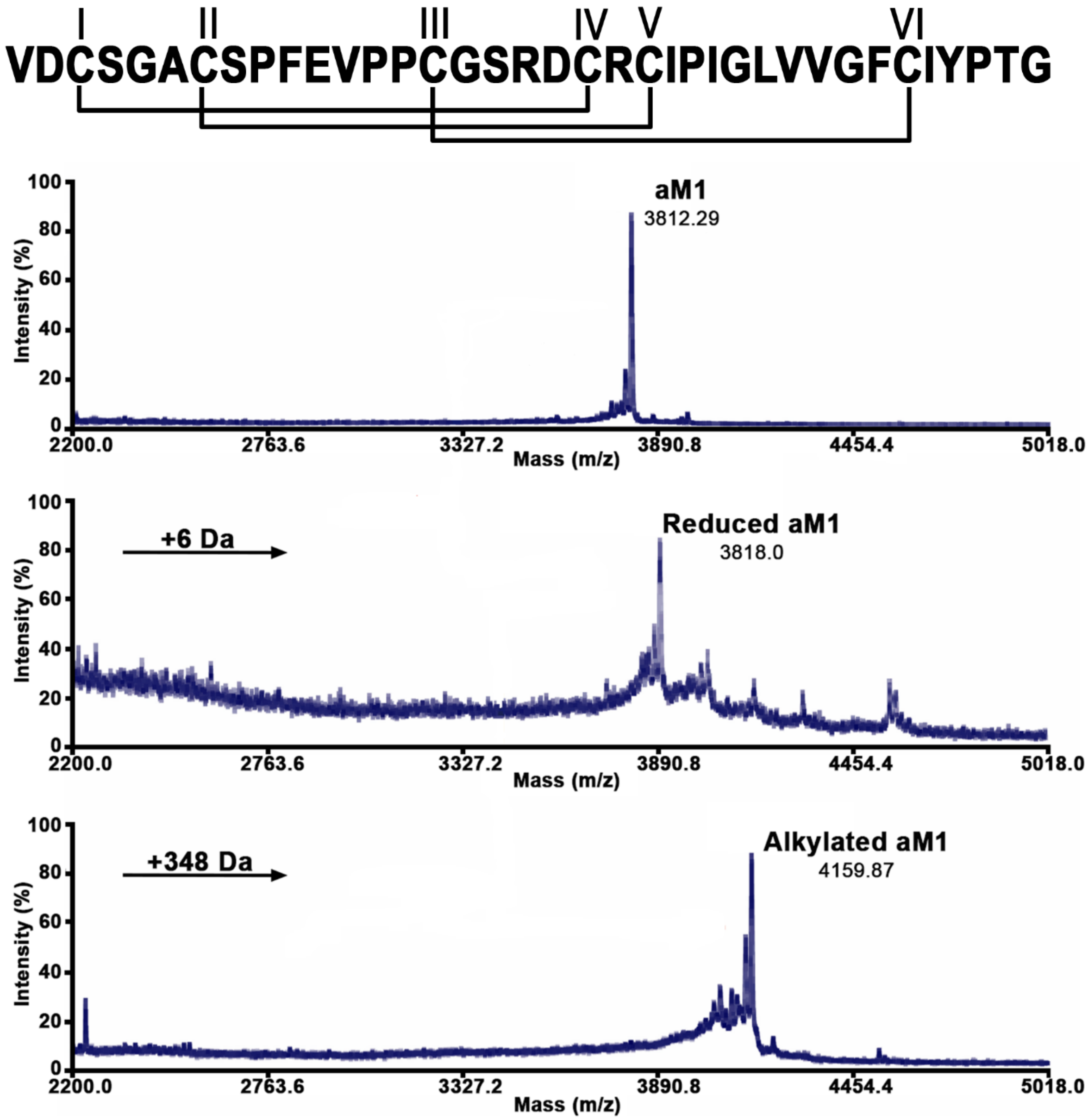

Figure S2: MALDI-TOF MS analysis of reduced and alkylated aM1. MS profiles of extracted and purified aM1 showed a mass of 3812.9 Da, and DTT-reduced aM1 showed a mass shift of 6 Da. S-alkylation by IAA increased the mass by 348 Da.

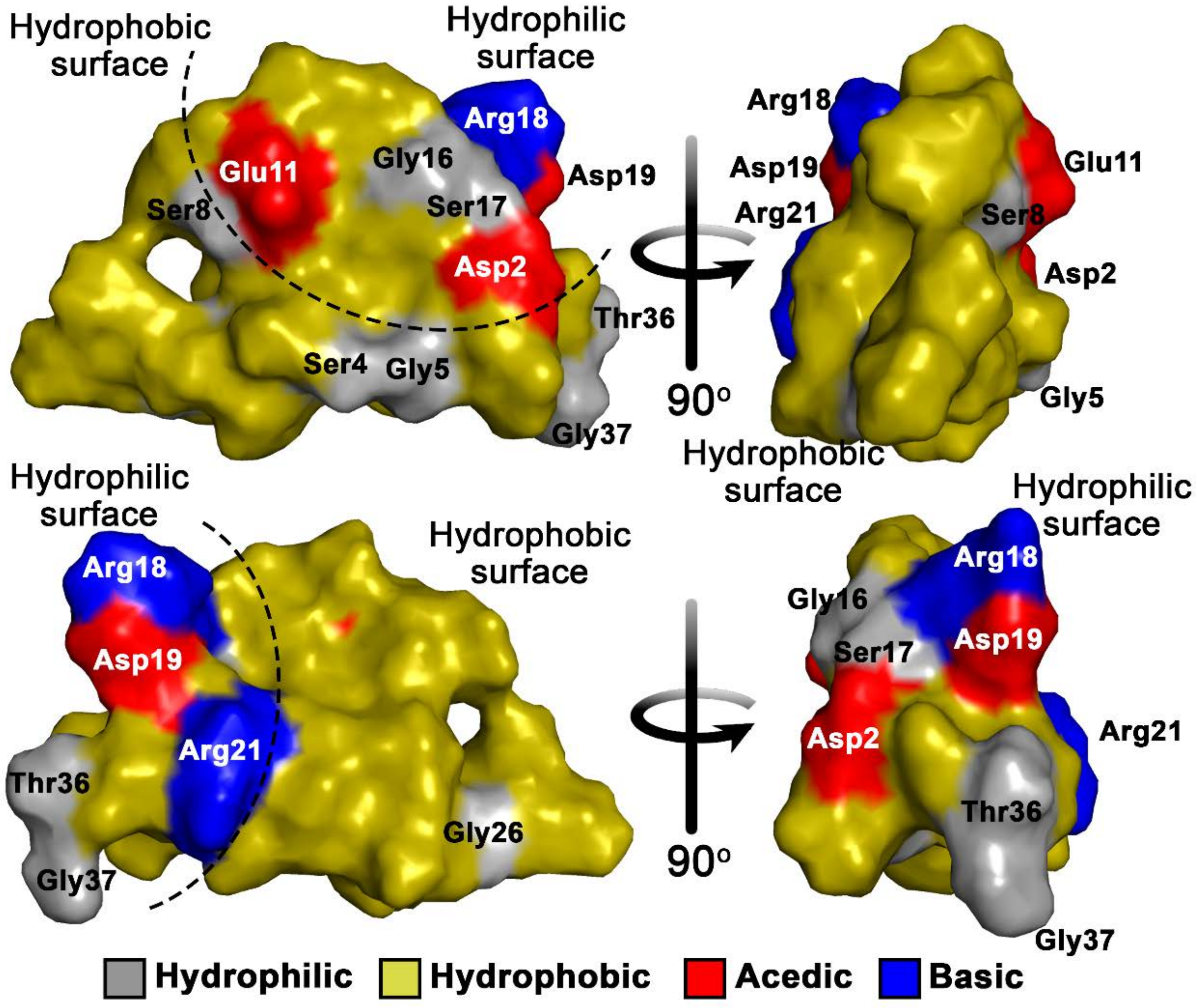

Figure S3: Surface properties of α-astratide aM1. The surface representation shows the localization of the hydrophobic and hydrophilic residues of aM1, respectively. The figures on the right are rotated 90° relative to those on the left. The figures on the bottom are turned 180° respective to the top.

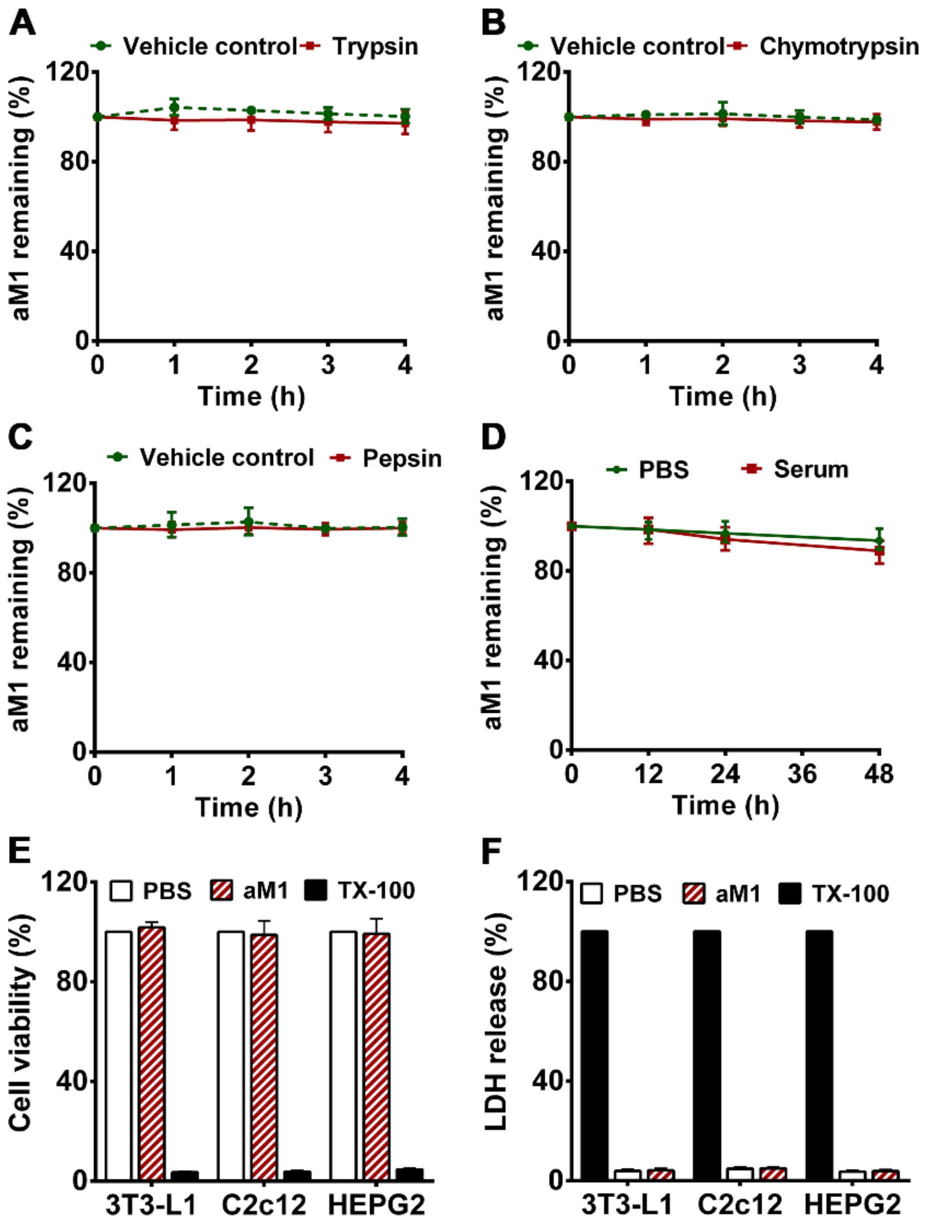

Figure S4: α-astratide aM1 is a highly stable, nontoxic, cystine-dense microprotein. A-C) Metabolic stability of aM1: aM1 was incubated in different gastrointestinal environments, including an alkaline environment (ammonium bicarbonate buffer, pH 8) with trypsin (A) and chymotrypsin (B) and simulated gastric fluid (0.1N HCl, pH 1.2) with pepsin for 4h at 37°C (C) and respective buffers were used as vehicle controls. Peptide quantification was performed using RP-HPLC, and data are shown as mean (± SD), calculated from experimental triplicates. D) Serum stability of aM1 was performed by incubating aM1 with human serum at 37 °C for 48h, and intact peptides were quantified using RP-HPLC. PBS was used as vehicle control. Data are presented as mean (± SD), calculated from individual experimental triplicates. E) MTT-based cell viability analysis of cells treated with or without100μM of aM1 for 24h and 1% Triton X 100 (TX-100) was used as a positive control for cell death. (n=3, unpaired multiple t-test with Holm-Šídák method-based correct for multiple comparisons); *p<0.05 compared to PBS control. F) LDH release-based cytotoxicity and plasma membrane damaging analysis of cells treated with or without 100μM of aM1 for 24h. (n=3, unpaired multiple t-test with Holm-Šídák method-based correct for multiple comparisons); *p<0.05 compared to TX-100.

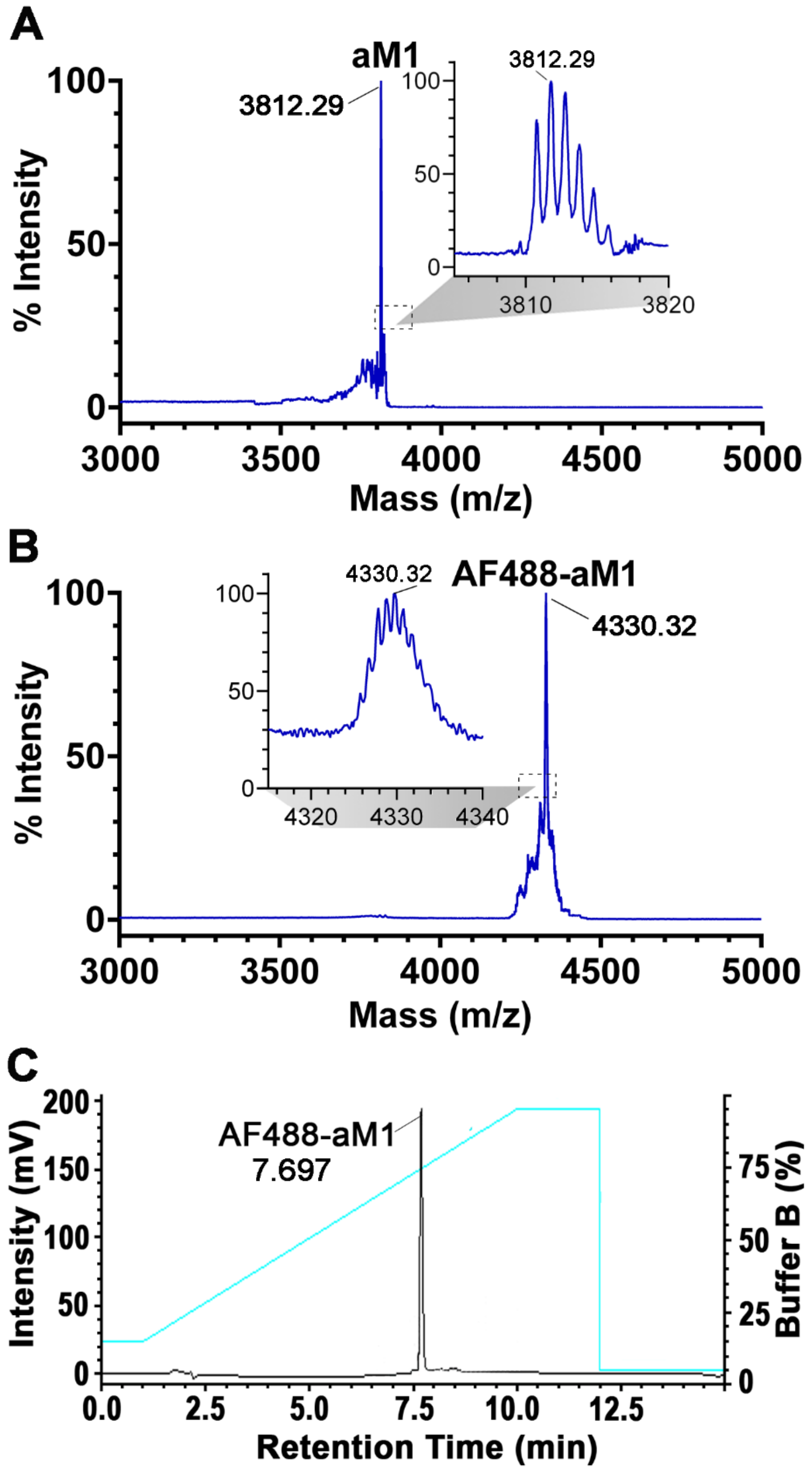

Figure S5: Analytical profiling of Alexa Fluor-488-labeled aM1 (AF488-aM1) after purifying by RP-HPLC. A-B) MALDI-TOF MS analysis of aM1 and AF488-aM1. C) HPLC analysis of purified AF488-aM1.

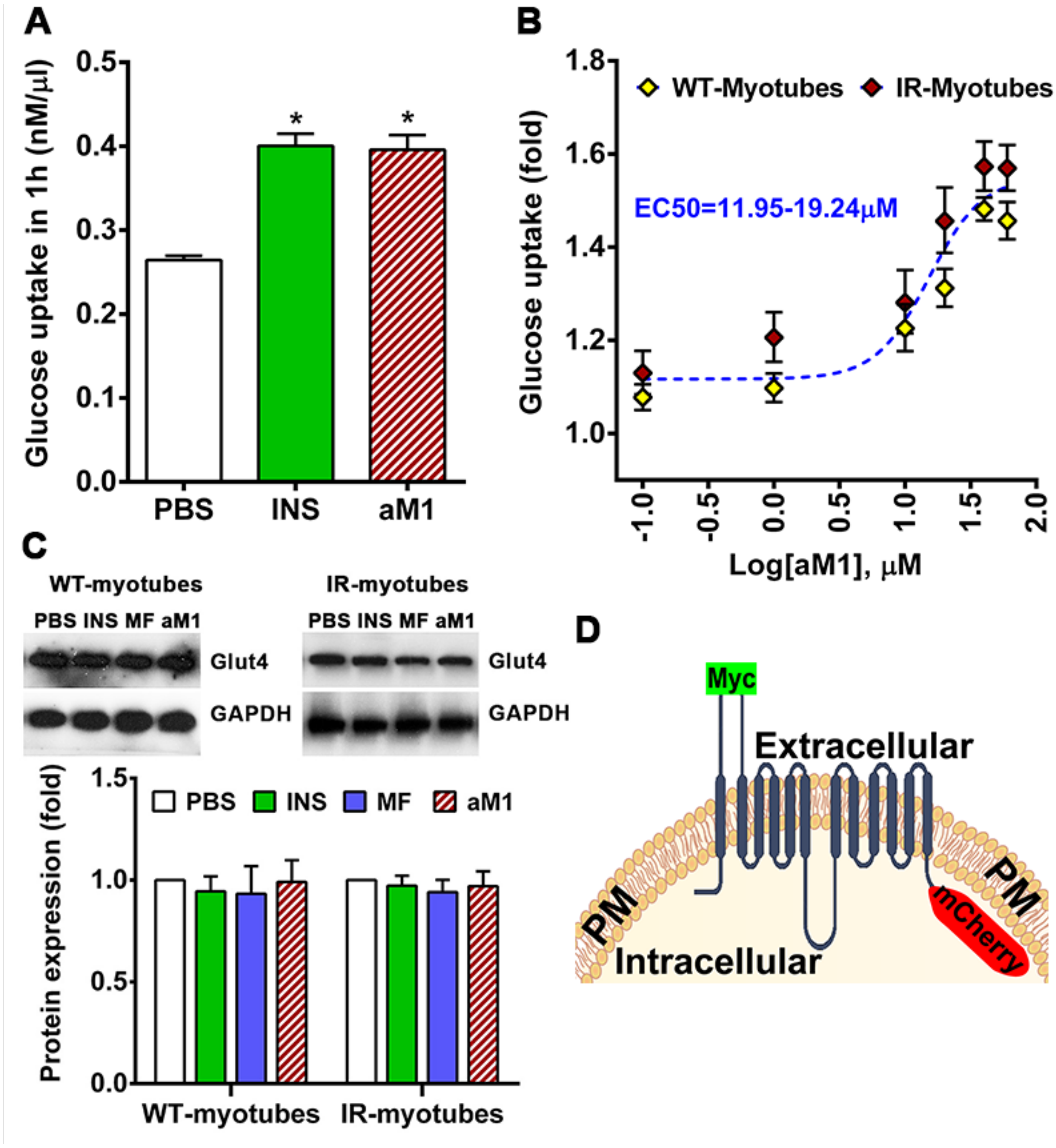

Figure S6: aM1-mediated glucose uptake is dose-dependent. A) C2C12 and HEPG2 cells were cultured with or without aM1 for 24h hours, and cellular glucose uptake was observed by fluorescence microscopy after staining with 2-NBDG. The relative quantification of 2-NBDG uptake was based on the fluorescence intensity in each image, and statistical significance was calculated for >50 individual images from three experimental replicates (analysis of variance (ANOVA) with Dunnett's multiple comparison test); *$p<0.05$ versus PBS-control. B) Dose-response curve of aM1.Wild-type (WT) and insulin-resistant (IR) C2C12-myotubes were treated with different doses (0–60μM) of aM1 for 24h, and a 2NBDG uptake assay was used to assess glucose uptake. Data are geometric means with 95% confidence intervals (CI) of the six independent experimental replicates. EC50 was calculated using Gaddum/Schild EC50 shift with 95%CI. C) Western blot analysis of Glut4 in wild-type (WT) and insulin-resistant (IR) C2C12-myotubes after insulin (INS), metformin (MF), and aM1 treatment. Glut4 expression was quantified using blot band intensity, and data in each group were normalized and showed as a fold of PBS control groups. Data showed mean ± SD (n=3, analysis of variance (ANOVA) with Tukey's multiple comparisons test). D) GLUT4 fusion protein used to detect and measure GLUT4 translocation and plasma membrane (PM) surface localization.

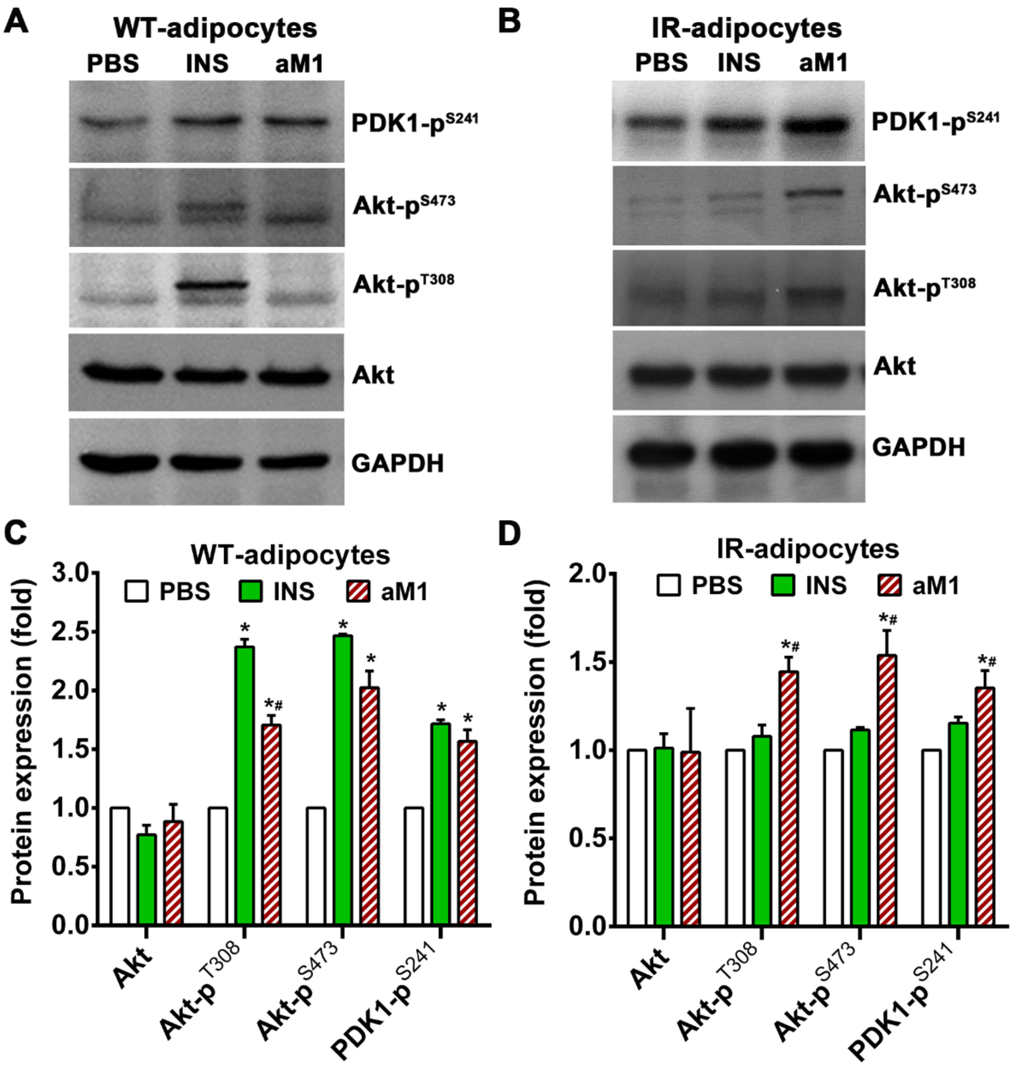

Figure S7: α-astratide aM1 activates PI3K/Akt pathway in 3T3-L1-adipocytes. Western blot analysis of the PI3K/Akt signaling in wild-type (WT) adipocytes (panel A) and insulin-resistant (IR) adipocytes (panel B) after treatment with PBS, insulin (INS), and aM1. Quantitative analysis of Western blot data on WT-3T3-L1-adipocytes (panel C) and IR-3T3-L1-adipocytes (panel D). Data in each group were normalized and expressed as a fold of PBS-treated respective control cells. Each bar represents mean ± SD (n=3, analysis of variance (ANOVA) with Tukey's multiple comparisons test). *$p<0.05$ versus PBS-control groups and $^{\#}p<0.05$ versus INS-treated groups.

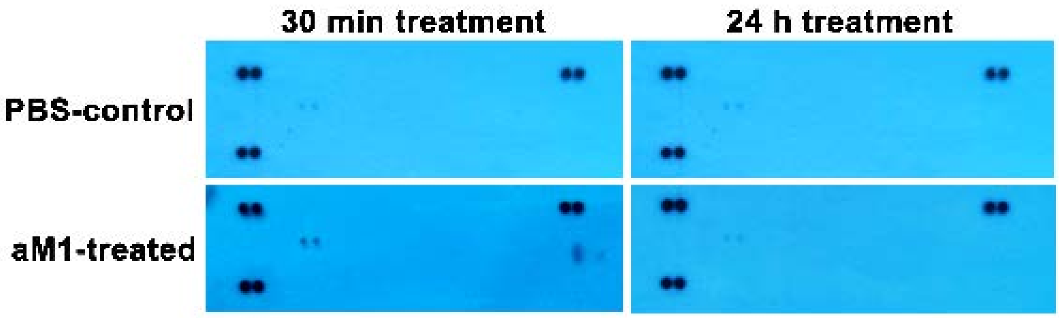

Figure S8: Phospho-RTK Array shows aM1 has no significant impact on RTK activation. Wild-type C2C12-myotubes were treated with or without 20μM aM1 for 30min and 24h, and RTK activation was assessed using Mouse Phospho-RTK Array Kit from R&D Systems (MN, USA). The array figure shows the phosphor-RTK levels in PBS-control and aM1-treated groups.

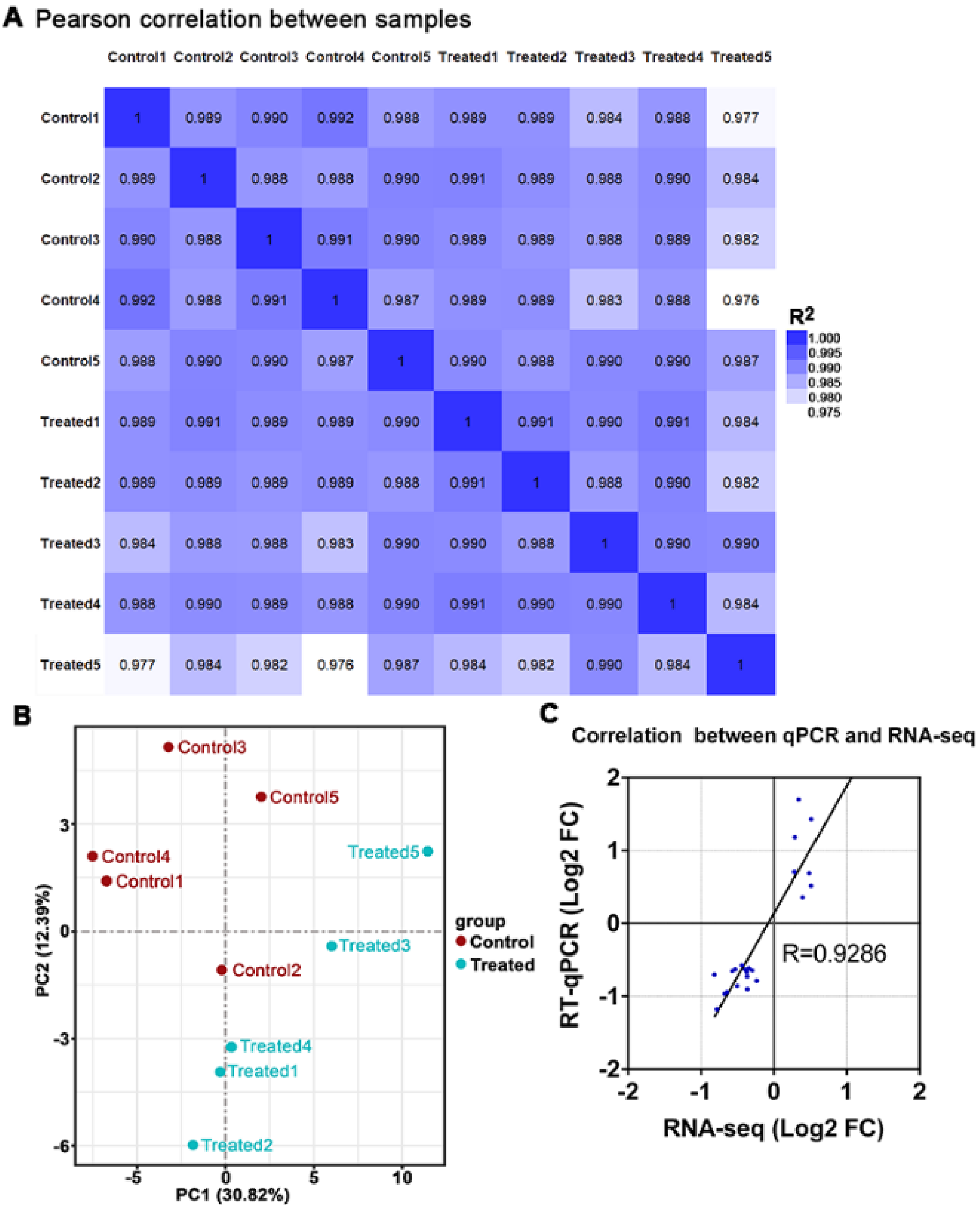

Figure S9: Transcriptome profiling of aM1-treated insulin resistant-C2C12 myotubes. A) Correlation coefficient matrix of control and aM1-treated samples. Five biological replicates were used for the RNA-seq analysis and R^2: Square of Pearson correlation coefficient (R). B) Principal component analysis (PCA) plots of the aM1-treated and control RNA-seq data (n=5). C) Correlation between the relative quantification of RNA-seq and RT-qPCR results. The value depicts the log_2 of the relative fold change (FC) between the control and aM1 treatment. Pearson's correlation coefficient (R) and regression line are presented.

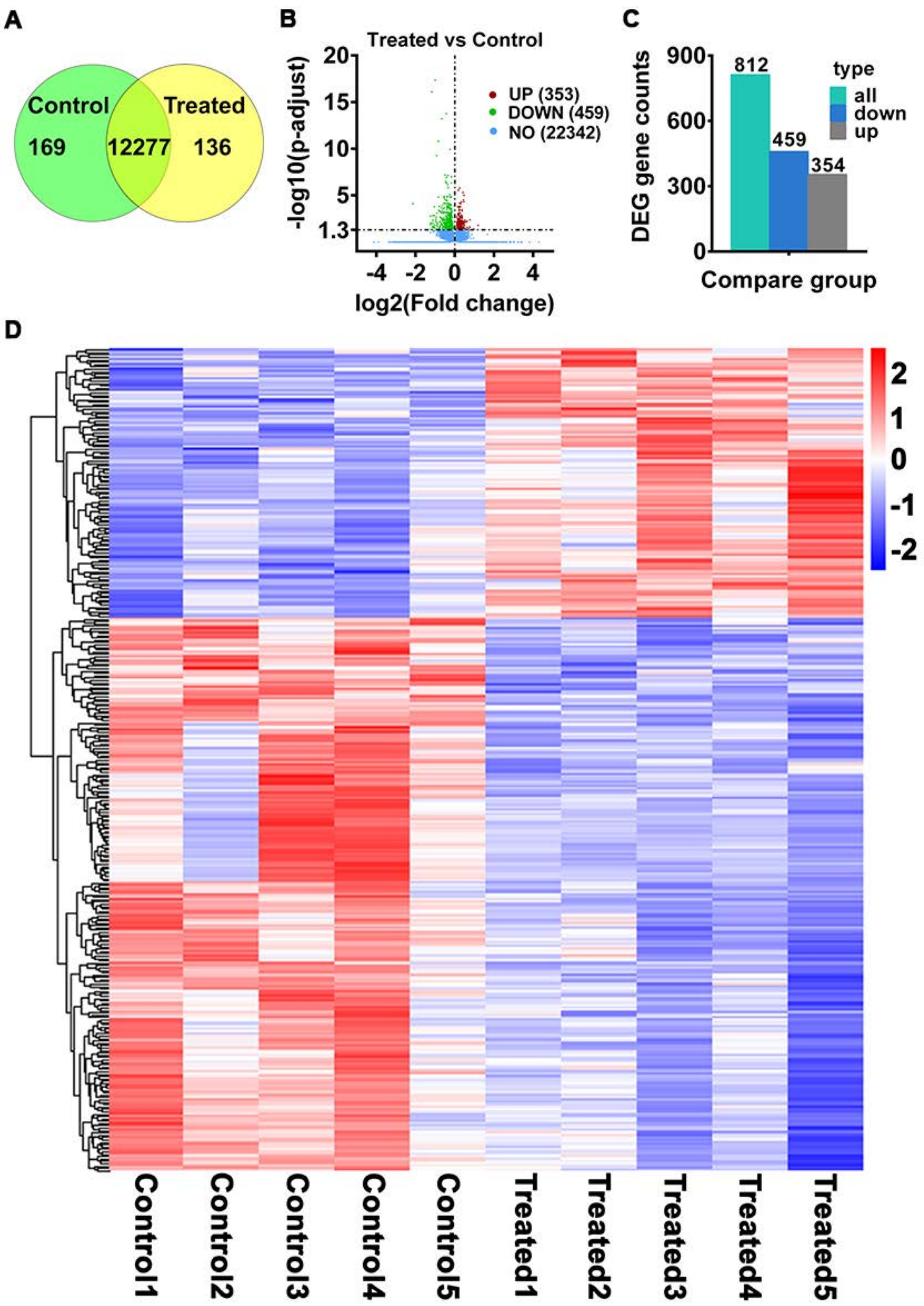

Figure S10: Transcriptome profiling shows aM1-mediated gene regulation in IR-C2C12 myotubes. A) Venn diagram showing differential gene expression among the control and aM1 treated groups. B) Volcano plot shows the overall distribution of differentially expressed genes in aM1-treated groups compared to control. C) The number of total differentially expressed genes (DEGs) and down-or up-regulated DEGs in aM1-treated samples. D) Hierarchical Clustering Heatmap. The overall results of FPKM cluster analysis clustered using the log2 (FPKM+1) value. Red indicates genes with high expression levels, and blue indicates genes with low expression levels. The red to blue indicates that log2 (FPKM+1) values were from large to small.

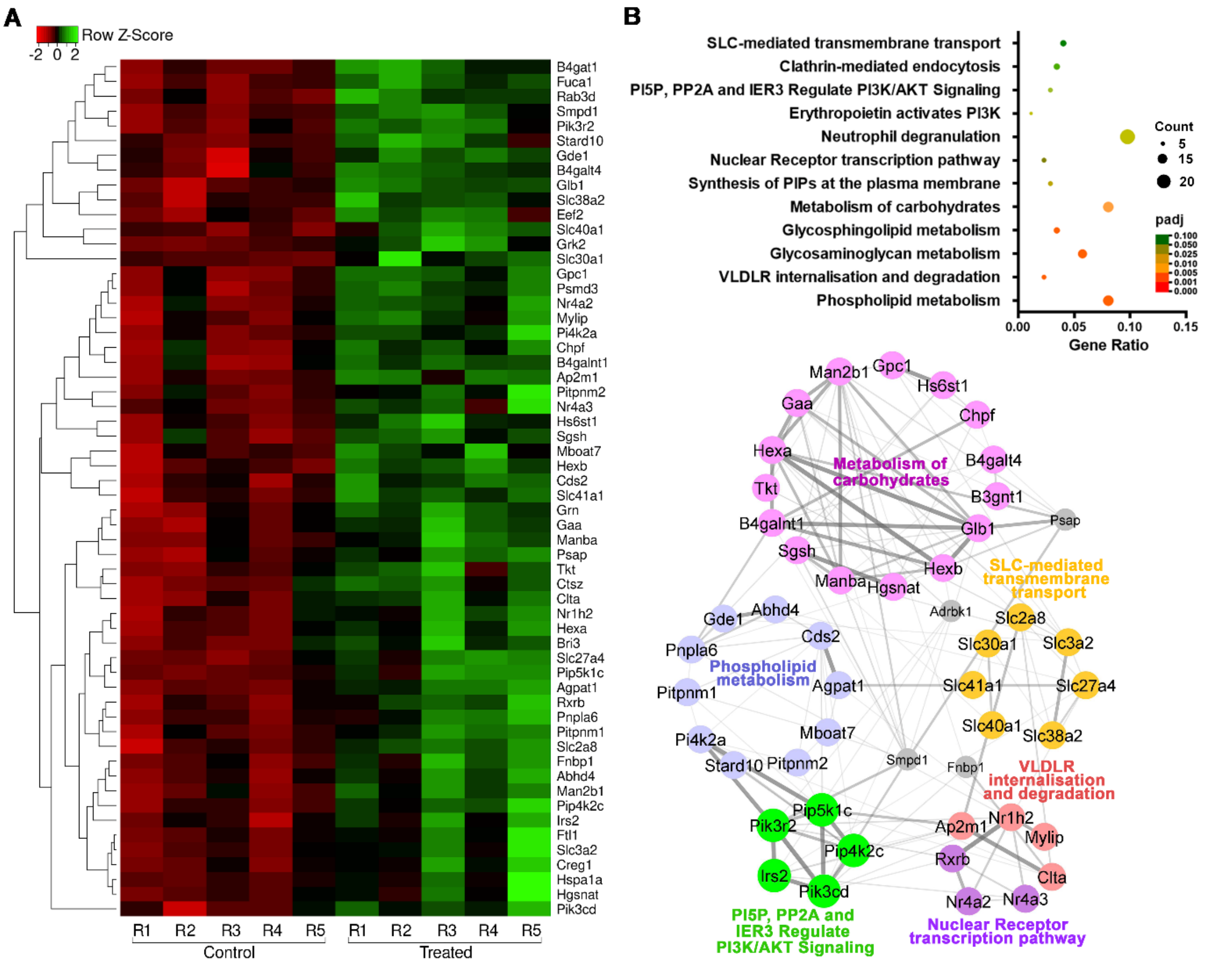

Figure S11: Differentially expressed genes (DEGs) analysis of the up-regulated genes in IR-C2C12 myotubes compared to aM1-treated-vs-control. (A) Heatmap for the up-regulated genes in the comparison of aM1-treated-vs-control; n=5.The color ranging from green to red indicates that gene expression values were larger to smaller. (B) Functional enrichment and pathway crosstalk of the up-regulated genes in the aM1-teated IR C2C12 myotubes.

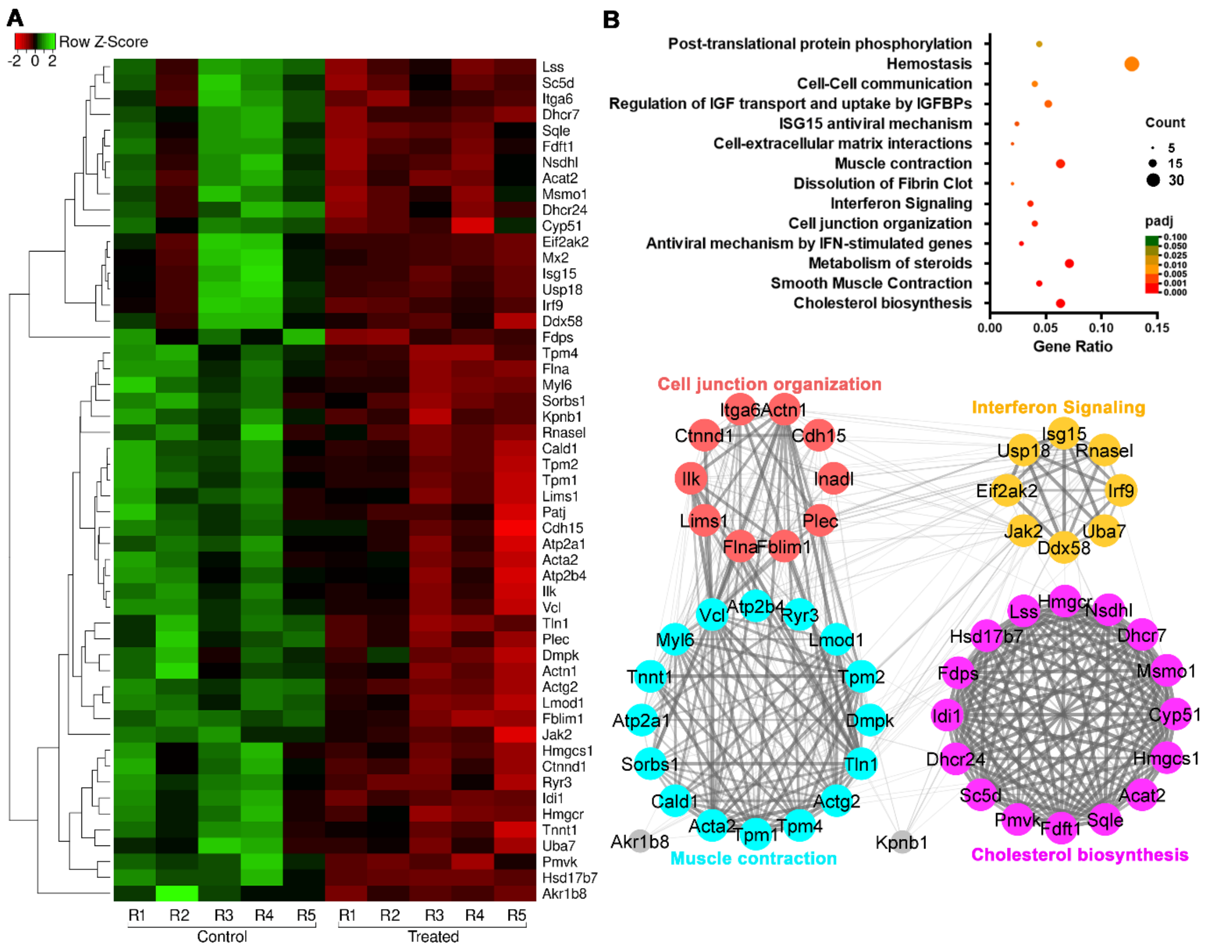

Figure S12: Differentially expressed genes (DEGs) analysis of the down-regulated genes in insulin resistant-C2C12 myotubes upon aM1 treatment. A) Heatmap for the down-regulated genes with opposite profiling compared to aM1-treated-vs-control; n=5. The color ranging from green to red indicates that gene expression values were larger to smaller. B) Functional enrichment and pathway crosstalk of down-regulated genes under aM1 treatment in IR- C2C12 myotubes.

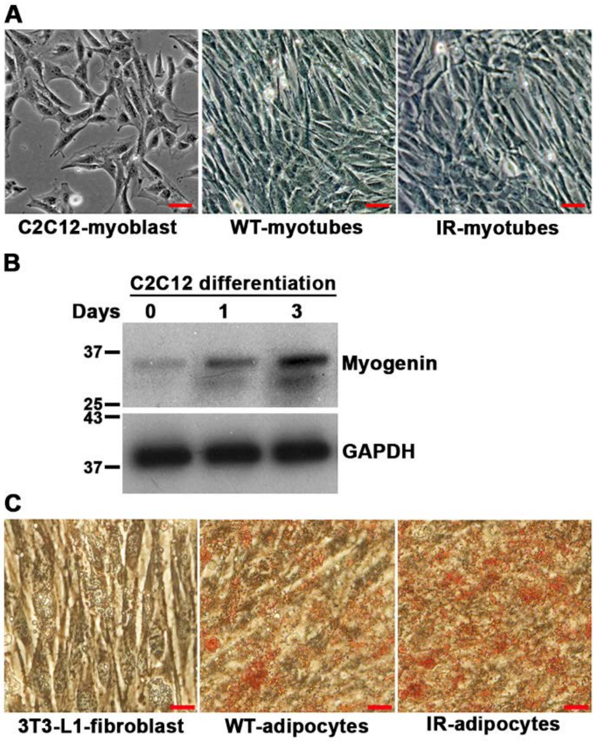

Figure S13: C2C12-myoblast and 3T3-L1-fibroblast differentiation and insulin-resistant phenotype. A) Microscopic images of C2C12 myoblast grown in complete growth medium (right panel). Wild-type (WT) myotubes (middle panel) were differentiated in differentiation media, and insulin-resistant (IR) myotubes (left panel) were differentiated in 100nM insulin-containing differentiation media for three days. Scale bar = 20µm. B) Myogenin expression indicates the C2C12-myoblast differentiation. C2C12 cells were serum starved, followed by exposure to a differentiated medium for three days, and myogenin expression was observed through western blot on day 0, day 1, and day 3. C) Microscopic images of 3T3-L1 fibroblasts grown in a complete growth medium (right panel). Wild-type (WT) (middle panel) and insulin-resistant (IR) (left panel) adipocyte phenotypes were generated by differentiating the 3T3-L1 cells, followed by maintaining them with or without insulin. Differentiation was confirmed by Oil Red staining. Scale bar = 20µm.

Full blot images

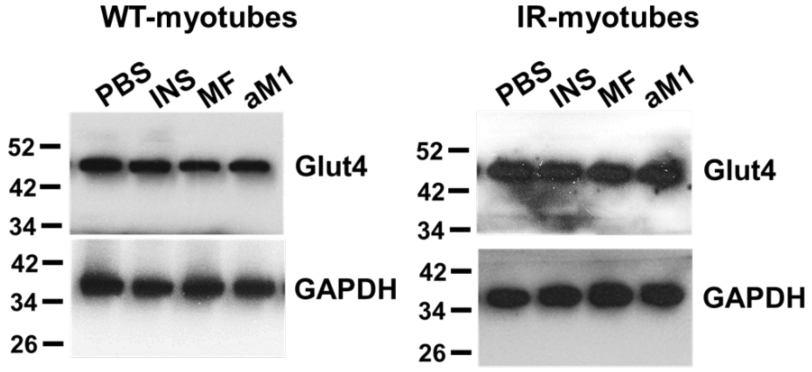

Full blot images: Western blot analysis in wild-type (WT) C2C12-myotubes and insulin-resistant (IR) C2C12-myotubes after treatment with PBS, insulin (INS), metformin (MF), and aM1. [Corresponding Figure S6].

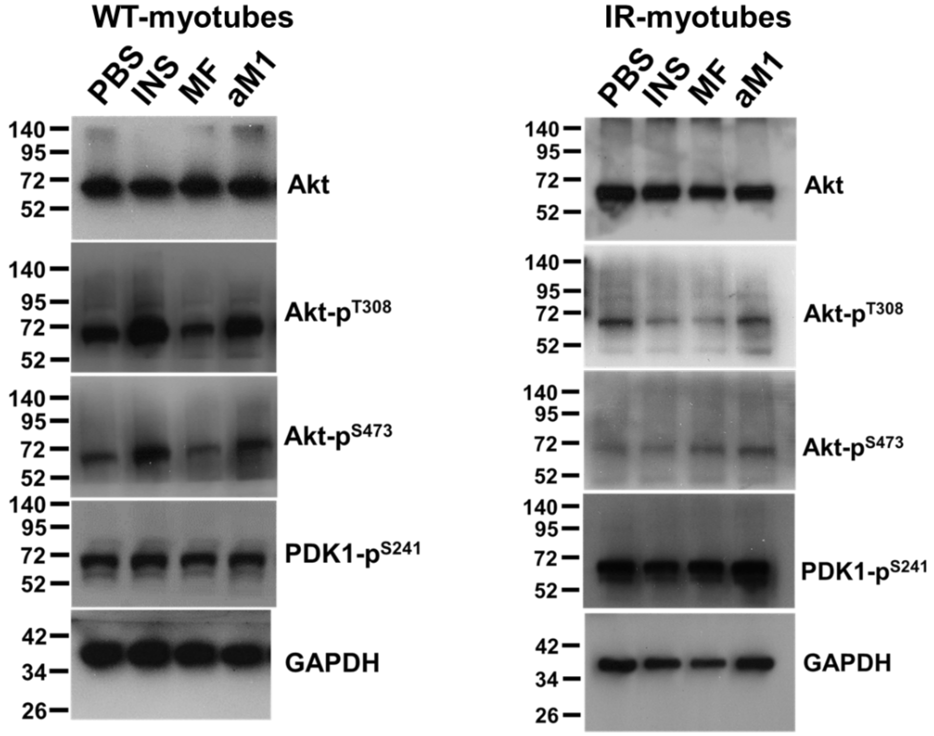

Full blot images. Western blot analysis in wild-type (WT) C2C12-myotubes and insulin-resistant (IR) C2C12-myotubes after treatment with PBS, insulin (INS), metformin (MF), and aM1. [Corresponding Figure 4].

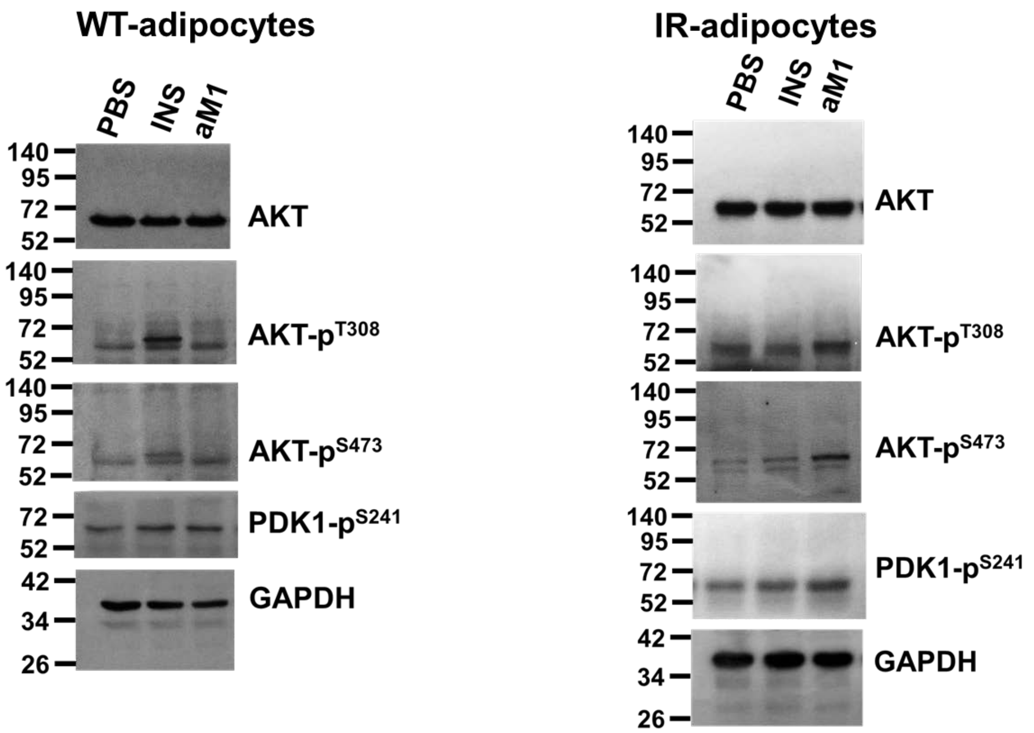

Full blot images. Western blot analysis in wild-type (WT) 3T3-L1-adipocytes and insulin-resistant (IR) 3T3-L1-adipocytes after treatment with PBS, insulin (INS), and aM1. [Corresponding Figure S7].

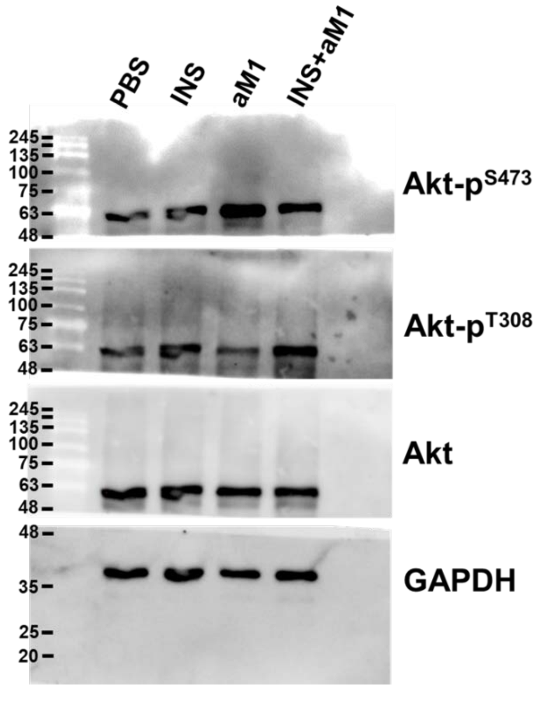

Full blot images. Western blot analysis in insulin-resistant (IR) C2C12-myotubes. Cells were pre-treated with PBS or aM1 for 24h and stimulated with Mock or insulin (INS) for 30min. [Corresponding Figure 6].

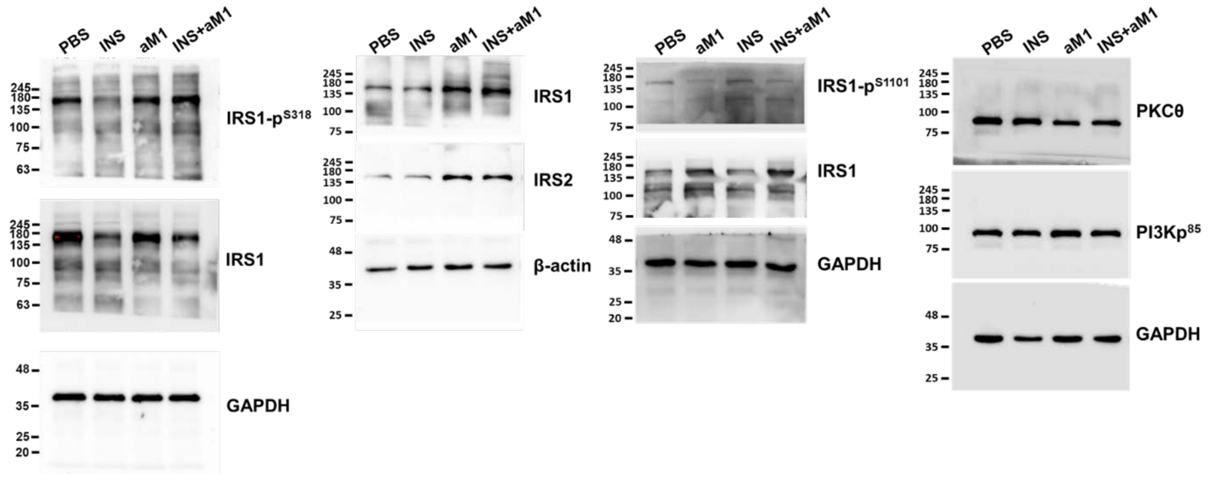

Full blot images. Western blot analysis in insulin-resistant (IR) C2C12-myotubes. Cells were pre-treated with PBS or aM1 for 24h and stimulated with Mock or insulin (INS) for 30min. [Corresponding Figure 9].
